# A metabolic switch toward lipid use in glycolytic muscle is an early pathologic event in a mouse model of amyotrophic lateral sclerosis

**DOI:** 10.15252/emmm.201404433

**Published:** 2015-03-27

**Authors:** Lavinia Palamiuc, Anna Schlagowski, Shyuan T Ngo, Aurelia Vernay, Sylvie Dirrig-Grosch, Alexandre Henriques, Anne-Laurence Boutillier, Joffrey Zoll, Andoni Echaniz-Laguna, Jean-Philippe Loeffler, Frédérique René

**Affiliations:** 1 INSERM, U1118, Mécanismes Centraux et Périphériques de la NeurodégénérescenceStrasbourg, France; 2 Université de Strasbourg, UMRS1118Strasbourg, France; 3 Equipe d'Accueil 3072, Mitochondrie, Stress oxydant et Protection Musculaire, Fédération de Médecine Translationelle de Strasbourg, Université de StrasbourgStrasbourg, France; 4 Service de Physiologie et d'Explorations Fonctionnelles, Pôle de Pathologie Thoracique Hôpitaux Universitaires, CHRU de StrasbourgStrasbourg, France; 5 School of Biomedical Sciences, The University of QueenslandSt Lucia, Qld, Australia; 6 University of Queensland Centre for Clinical Research, The University of QueenslandHerston, Qld, Australia; 7 UMR7364 Laboratoire de Neurosciences Cognitives et Adaptatives, Faculté de Psychologie, Université de Strasbourg-CNRS, GDR CNRS 2905Strasbourg, France; 8 Département de Neurologie, Hôpital de Hautepierre, Hôpitaux Universitaires de StrasbourgStrasbourg, France

**Keywords:** amyotrophic lateral sclerosis, exercise, glucose, lipids, muscle

## Abstract

Amyotrophic lateral sclerosis (ALS) is the most common fatal motor neuron disease in adults. Numerous studies indicate that ALS is a systemic disease that affects whole body physiology and metabolic homeostasis. Using a mouse model of the disease (SOD1^G86R^), we investigated muscle physiology and motor behavior with respect to muscle metabolic capacity. We found that at 65 days of age, an age described as asymptomatic, SOD1^G86R^ mice presented with improved endurance capacity associated with an early inhibition in the capacity for glycolytic muscle to use glucose as a source of energy and a switch in fuel preference toward lipids. Indeed, in glycolytic muscles we showed progressive induction of pyruvate dehydrogenase kinase 4 expression. Phosphofructokinase 1 was inhibited, and the expression of lipid handling molecules was increased. This mechanism represents a chronic pathologic alteration in muscle metabolism that is exacerbated with disease progression. Further, inhibition of pyruvate dehydrogenase kinase 4 activity with dichloroacetate delayed symptom onset while improving mitochondrial dysfunction and ameliorating muscle denervation. In this study, we provide the first molecular basis for the particular sensitivity of glycolytic muscles to ALS pathology.

## Introduction

Amyotrophic lateral sclerosis (ALS) is the most common adult motor neuron disease. Although historically defined as an age-related neurodegenerative disease specifically affecting upper and lower motor neurons, 145 years of research has now identified for ALS to be unquestionably more complex than initially described. In recent years, several non-neuronal cells including microglia (Boillée *et al*, 2006), astrocytes (Yamanaka *et al*, 2008), and skeletal muscle (Wong & Martin, 2010) have been proposed to influence the course of the disease. Moreover, mutations in an increasing number of genes have been associated with familial and sporadic forms of ALS (Chen *et al*, 2013). In accordance with this genetic heterogeneity, ALS has recently been redefined as a ‘syndrome’, regrouping complex and diverse pathophysiological and clinical manifestations that transgress the motor system (Andersen & Al-Chalabi, 2011; Hardiman *et al*, 2011).

Numerous studies indicate that ALS is a systemic disease that affects whole body physiology and energy homeostasis. In 2001, a systematic analysis of energy expenditure rates in a population of sporadic ALS patients revealed that two-thirds of these patients were hypermetabolic (Desport *et al*, 2001). These observations were further confirmed and extended to familial ALS cases in follow-up reports (Desport *et al*, 2005; Bouteloup *et al*, 2009). ALS patients as well as mouse models of ALS present with weight loss and reduced fat mass, altered glucose and lipid handling, and increased resting energy expenditure (Dupuis *et al*, 2011). In mice, reduced lipid stores and increased resting energy expenditure precede motor symptoms (Dupuis *et al*, 2004). Interestingly, the burden of these metabolic alterations appears to affect the neurodegenerative process. Indeed, metabolic alterations correlate with duration of survival, and clinical data suggest that an imbalance in energy metabolism has a negative impact on the pathogenic process (Desport *et al*, 1999; Jawaid *et al*, 2010). Moreover, increased dietary lipid content offers neuroprotection and extends survival in mouse models of ALS (Dupuis *et al*, 2004; Mattson *et al*, 2007), while restricting calorie intake exacerbated motor symptoms in ALS mice (Pedersen & Mattson, 1999). Although the origin of metabolic dysfunction in ALS remains unclear, many of the metabolic modifications observed at the systemic level in ALS patients and related mouse models imply that skeletal muscle contributes to ALS progression.

At rest, skeletal muscle accounts for 20–30% of the total energy expenditure. Given that skeletal muscle plays a critical role in maintaining metabolic homeostasis, altered energy balance in this tissue over extended periods of time may represent a risk factor for the development of ALS. Interestingly, thorough mapping of neuromuscular junction (NMJ) denervation patterns in the SOD1^G93A^ mouse model of ALS revealed increased susceptibility of glycolytic fibers to undergo denervation. The loss of fast fatigable motor units composed of large motor neurons innervating type IIb fast glycolytic fibers before any apparent motor symptoms (Frey *et al*, 2000; Hegedus *et al*, 2008) suggests that the metabolic signature of glycolytic fibers may predispose them to NMJ dismantlement, one of the first pathologic events in the course of the disease (Fischer *et al*, 2004). Indeed, we have previously shown that promotion of muscle-specific hypermetabolism is sufficient to induce some hallmarks of ALS including destruction of the neuromuscular junction (NMJ) and the death of motor neurons (Dupuis *et al*, 2009). Collectively, these data suggest that metabolic changes in skeletal muscle could contribute to early destabilization of the NMJ in ALS.

In this study, we investigated early muscle metabolic alterations that might account for the metabolic imbalance and early NMJ denervation observed in SOD1^G86R^ mice. We report an early alteration in muscle metabolic homeostasis in SOD1^G86R^ mice. The switch toward lipid use in glycolytic muscle precedes NMJ denervation detected by EMG and the increase in expression of denervation markers. By administering dichloroacetate (DCA) to SOD1^G86R^ mice, we reversed metabolic imbalance, improved metabolic function, and normalized the expression of denervation markers in SOD1^G86R^ mice. Our results reveal an early metabolic pathology in skeletal muscle that contributes to the severity of the disease and adds new insight into the already complex ALS syndrome.

## Results

### Asymptomatic SOD1^G86R^ mice have increased aerobic capacity

**Figure 1:**
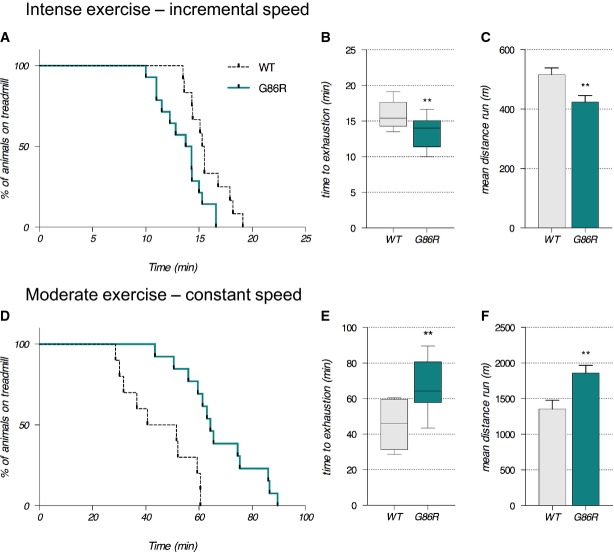
65-day-old SOD1^G86R^ mice have improved performance during endurance exercise

During ALS disease progression, a progressive shift in skeletal muscle fiber subtype from fast twitch to slow twitch would presumably result in a concomitant shift toward more oxidative metabolism (Dengler *et al*, 1990; Frey *et al*, 2000; Pun *et al*, 2006; Deforges *et al*, 2009). Thus, we aimed to determine whether SOD1^G86R^ mice would have improved capacity to support endurance exercise. We compared anaerobic versus aerobic capacities of 65-day-old asymptomatic SOD1^G86R^ mice using two exercise paradigms on a treadmill apparatus: an intense anaerobic exercise that recruits glycolytic fast-twitch fibers (Fig1A–C) and a low-intensity endurance exercise that mobilizes slow-twitch oxidative muscle fibers (Fig1D–F). As shown in Fig1A, when treadmill speed was progressively increased in the intense anaerobic exercise paradigm, 65-day-old SOD1^G86R^ mice had significantly poorer performance than their WT littermates; SOD1^G86R^ mice ran on the treadmill for an average of 13.50 min (median at 14.33), while their WT littermates spent an average of 15.78 min (median at 15.50) on the treadmill (Fig1B). Accordingly, SOD1^G86R^ mice ran a total distance that was 17.6% shorter than the distance covered by their WT littermates (Fig1C). By contrast, when challenged with a moderate exercise paradigm that is representative of an endurance workout (Fig1D–F), SOD1^G86R^ mice proved to have significantly greater endurance than their WT littermates (Fig1D). In response to moderate exercise, SOD1^G86R^ mice ran an average of 67.34 min (median at 65.43) before reaching exhaustion, while their WT littermates ran an average of 46.10 min (median at 59.33) prior to exhaustion (Fig1D). Indeed, the resistance of SOD1^G86R^ mice to reach exhaustion when subjected to this exercise paradigm resulted in them running a total distance that was 36% greater than the distance covered by their WT littermates (Fig1E).

A–F (A–C) Exercise performance on a treadmill apparatus using an intense exercise (incremental speed paradigm) that relies solely on anaerobic metabolism. (D–F) Exercise performance on a treadmill apparatus using a moderate exercise (constant sub-maximal speed) that relies on the recruitment of aerobic metabolism and that is representative of endurance exercise. (A, D) Kaplan–Meier curves representing values corresponding to the percentage of mice still running at a given time point. (B, E) Median values of the time that mice ran are represented in the min to max graphical representation; 15.5 min for wild-type (WT) and 14.33 min for SOD1^G86R^ in (B); 59.33 min for WT and 65.43 min for SOD1^G86R^ in (E). (C, F) Mean distance run ± SEM by WT and SOD1^G86R^ mice in (C) and by WT and SOD1^G86R^ mice in (F). Data information: G86R versus WT, *P* = 0.017 in (A), **P = 0.0078 in (B), **P = 0.008 in (C) and *P* = 0.036 in (D) (Mantel–Cox test), **P = 0.0078 in (E), and **P = 0.0061 in (F) (Student's *t*-test), *n* = 10 and 13 for WT and SOD1^G86R^, respectively.

**Figure 2:**
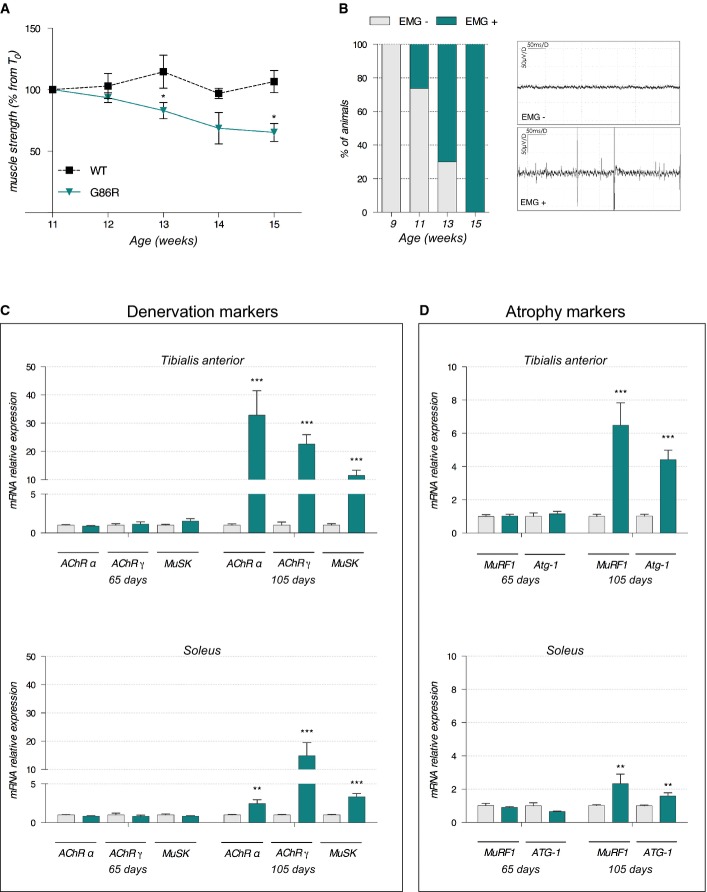
Decreased grip strength, increased denervation, and increased expression of denervation and atrophy markers throughout disease progression in SOD1^G86R^ mice

Given that skeletal muscle possesses the capacity to adapt metabolically to physical and functional challenges (Constable *et al*, 1987; Bassel-Duby & Olson, 2006), the enhanced aerobic capacity and poor anaerobic capacity of SOD1^G86R^ mice might occur as a consequence of muscle denervation. In this study, we assessed the expression of *AChRα* and *AChRγ*, two subunits of the nicotinic acetylcholine receptor, and *Musk*, a muscle-specific kinase, all of which are rapidly overexpressed after denervation or when muscle electrical activity is absent (Duclert & Changeux, 1995; Valenzuela *et al*, 1995). However, at 65 days of age SOD1^G86R^ mice did not present with any sign of denervation regardless of the metabolic capacity of muscle; muscle grip strength was comparable to that of WT littermates (Fig2A), EMG profiles were normal (Fig2B), and the mRNA expression of molecular markers of denervation (Fig2C) and atrophy (Fig2D) in glycolytic *tibialis anterior* (TA) or oxidative *soleus* muscles was similar to WT levels. By contrast, at 105 days when SOD1^G86R^ mice are paralyzed and grip strength is decreased (Fig2A), all SOD1^G86R^ presented with increased spontaneous activity on their EMG profiles (Fig2B). Moreover, induction of denervation and atrophy markers was more pronounced in the TA than in the *soleus* (Fig2C and D). These data support previous observations that in ALS, glycolytic muscles are more severely affected by disease pathology than oxidative muscles (Dengler *et al*, 1990; Frey *et al*, 2000; Pun *et al*, 2006). We therefore focused our subsequent analysis on the glycolytic TA.

Grip test was performed to measure mouse muscle strength at different time points during disease progression. Graph represents the mean of percentage from initial force measured at 11 weeks ± SEM. At 13 weeks, *P =* *0.03; at 14 weeks, *P *=* *0.06; at 15 weeks, *P =* *0.04 (*n* = 8 for WT and SOD1^G86R^, respectively, at 11–15 weeks, two-way ANOVA followed by Fisher's LSD *post hoc* test).

Electromyography (EMG) recordings were performed weekly during disease development, starting from 9 weeks of age. Spontaneous electrical activity was considered as positive EMG only for peak-to-peak amplitudes > 50 μV. Left: The percentage of mice presenting with positive EMG in each age group are represented. Right: Representative examples of negative (EMG^−^) and positive (EMG^+^) electromyography profiles (*n* = 10 for WT and SOD1^G86R^, respectively, at 9–13 weeks and *n* = 8 and 7 for WT and SOD1^G86R^, respectively, at 15 weeks).

Relative mRNA levels of denervation markers AChR (subunits α and γ) and MuSK were evaluated by qPCR at the indicated ages (65 and 105 days) in *tibialis anterior* (upper panels) and *soleus* (lower panels) of WT and SOD1^G86R^ mice. Graphs represent mean fold change ± SEM from age-matched WT (*n* > 5). ***P-values versus WT: < 0.0001 for *AChRα*, *AChRγ,* and *MuSK* in *tibialis anterior*; and **P-values versus WT: 0.001 for *AChRα*, and ***P-values versus WT: 0.0007 for *AChRγ*, 0.0002 for *MuSK* in *soleus* (*n*=7 and 8 for WT and SOD1^G86R^, respectively, at 65 days; *n* = 5 and 6 for WT and SOD1^G86R^, respectively, at 105 days; two-way ANOVA followed by Fisher's LSD *post hoc* test).

Relative mRNA levels of muscle atrophy markers MuRF1 and Atg-1 were measured by qPCR at the indicated ages (65 and 105 days) in *tibialis anterior* (upper panels) and *soleus* (lower panels). Graphs represent mean fold change ± SEM from age-matched WT. ***P-values versus WT: < 0.0001 for *MuRF1* and *Atg-1* in *tibialis anterior*; and **P-values versus WT: 0.007 for *MuRF1* and *P *=* *0.006 for *Atg-1* in *soleus* (*n* = 7 and 8 for WT and SOD1^G86R^, respectively, at 65 days, *n* = 5 and 6 for WT and SOD1^G86R^, respectively, at 105 days, two-way ANOVA followed by Fisher's LSD *post hoc* test).

### Muscle glucose metabolism is inhibited in asymptomatic SOD1^G86R^ mice

Glycolysis is the only metabolic pathway that provides a source of high-energy substrates for anaerobic exercise. Given that impaired glycolytic performance in SOD1^G86R^ mice cannot be explained by denervation or atrophy, we hypothesized that reduced anaerobic performance was due to defective glucose utilization. In order to verify whether glucose metabolism is altered in SOD1^G86R^ mice, we first analyzed their response to glucose and insulin. The glucose tolerance test (Supplementary Fig S1A) showed that at 65 days, SOD1^G86R^ mice presented with higher blood glucose at 30 and 45 min when compared to WT littermates, suggesting that initial glucose clearance is delayed in SOD1^G86R^ mice. However, after 120 min blood glucose levels were similar between WT and SOD1^G86R^ mice, suggesting that there may be increased glucose uptake between 30 and 120 min in SOD1^G86R^ mice. The response to insulin was also delayed in SOD1^G86R^ mice when compared to WT littermates (Supplementary Fig S1B). These data show an alteration in glucose handling in SOD1^G86R^ mice at the asymptomatic stage of disease.

**Figure 3:**
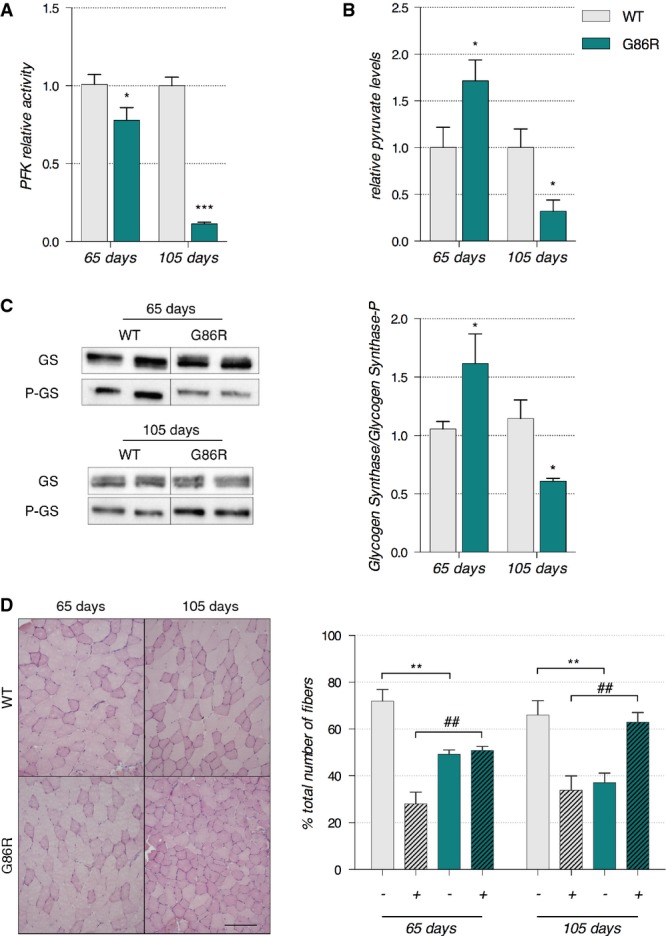
Phosphofructokinase 1 is inhibited in glycolytic muscle and glucose is rerouted toward glycogen stores

To determine whether altered glucose handling occurred at the level of skeletal muscle, we assessed the activity of phosphofructokinase 1 (PFK1), the rate-limiting enzyme of glycolysis, in TA cytosolic homogenates (Fig3A). When compared to WT littermates, SOD1^G86R^ mice had a significant 23% reduction in PFK1 enzymatic activity at 65 days of age. By the end stage of disease (105 days of age), PFK1 activity was reduced by 88.8% in SOD1^G86R^ mice when compared to WT littermates. This was accompanied by a 5-fold down-regulation in the expression of *Pfk1* mRNA in SOD1^G86R^ mice (Supplementary Fig S1C). Interestingly, a 4-fold down-regulation in the expression of *Pfk1* mRNA was also observed in SOD1^G93A^ mice, another ALS mouse model, at the end stage of disease (Supplementary Fig S2B).

Enzymatic activity of phosphofructokinase in whole *tibialis anterior* muscle cytosolic homogenates is expressed as mean fold change ± SEM from age-matched WT at 65 days of age *P =* *0.016 (WT *n* = 6, SOD1^G86R^ *n* = 7) and 105 days of age ***P <* *0.0001 (*n* = 5/genotype), two-way ANOVA followed by Fisher's LSD *post hoc* test. Data shown are representative of two independent experiments having similar results.

Pyruvate was measured in whole *tibialis anterior* muscle tissue homogenates. The mean fold change ± SEM compared to age-matched WT are represented with *P =* *0.019 at 65 days of age (*n* = 5/genotype) and *P =* *0.041 at 105 days of age (*n* = 4/genotype), two-way ANOVA followed by Fisher's LSD *post hoc* test. Data shown are representative of two independent experiments having similar results.

Left: Representative Western blot showing glycogen synthase and phosphorylated glycogen synthase levels. Right: Quantification represents the mean ratio of optical densities of glycogen synthase/P-glycogen synthase ± SEM. At 65 days of age *n* = 6/genotype, *P =* *0.0162, and at 105 days of age *n* = 5/genotype, *P =* *0.0163, two-way ANOVA followed by Fisher's LSD *post hoc* test.

Left: Representative microphotographs of PAS staining from WT and SOD1^G86R^ *tibialis anterior* cross-sections at 65 and 105 days of age showing glycogen-negative (light pink) and glycogen-positive (dark pink) fibers. Scale bar: 200 μm. Right: Quantification of glycogen-negative (−) and glycogen-positive (+) fibers at 65 and 105 days of age. For (−) fibers: **P-values versus WT: 0.0026 at 65 days and 0.0074 at 105 days, for (+) fibers: ^##^*P*-values versus WT: 0.0025 at 65 days and 0.0073 at 105 days (*n* = 5/genotype at 65 days and *n* = 4/genotype at 105 days, two-way ANOVA followed by Fisher's LSD *post hoc* test).

Glycolysis is regulated by a number of upstream and downstream components. Pyruvate is the end product of glycolysis and a downstream component in the glycolytic pathway that inhibits muscle PFK1 while glycogen synthase is a key enzyme that converts glucose to glycogen. Thus, we assessed pyruvate levels, glycogen synthase activity, and glycogen accumulation in the TA of SOD1^G86R^ mice and WT littermates at the asymptomatic and symptomatic stages of disease. Pyruvate was 1.7-fold more concentrated in 65-day-old SOD1^G86R^ mice than in age-matched WT littermates (Fig3B). By 105 days of age, symptomatic SOD1^G86R^ mice had a significant reduction in pyruvate levels when compared to WT littermates. Changes in the expression of pyruvate levels in SOD1^G86R^ mice occurred concurrently with altered activity of glycogen synthase and altered glycogen accumulation. At 65 days of age, SOD1^G86R^ mice had decreased phosphorylation of glycogen synthase (Fig3C) and a corresponding increase in glycogen accumulation (Fig3D). By contrast, glycogen synthase phosphorylation in SOD1^G86R^ mice at 105 days of age was dramatically increased, indicating the inhibition of its activity. The increased synthesis of glycogen and its subsequent accumulation was quantified in transverse sections of TA stained by periodic acid-Schiff (PAS) (Fig3D).

### Pyruvate dehydrogenase kinase 4 up-regulation in glycolytic muscle tissue is an early event

**Figure 4:**
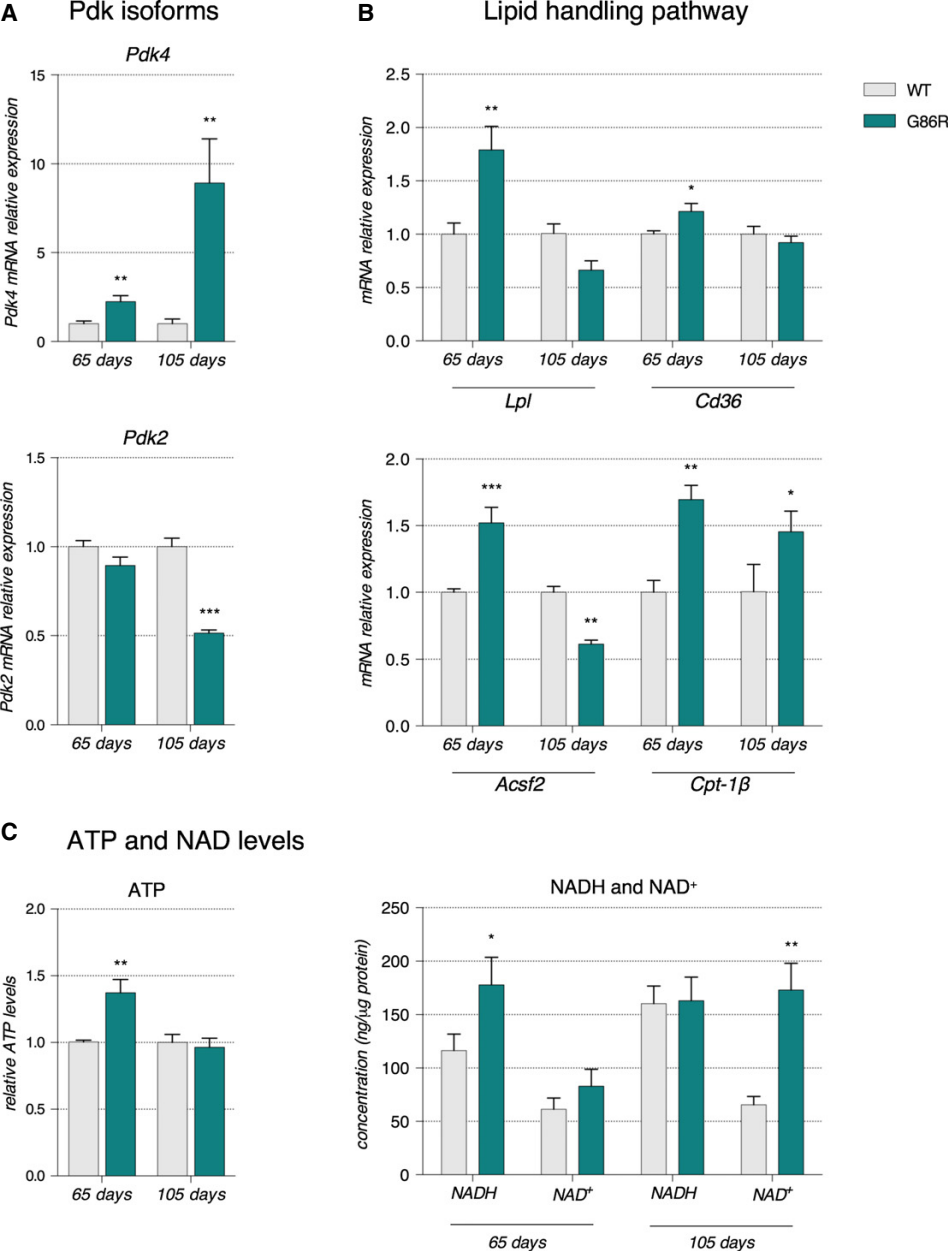
Altered fuel preference in glycolytic muscle of 65-day-old asymptomatic SOD1^G86R^ mice

In skeletal muscle, pyruvate levels are primarily governed by pyruvate dehydrogenase complex (PDH) activity, which itself is inhibited when phosphorylated by pyruvate dehydrogenase kinase 4 (PDK4). Thus, we assessed the expression levels of *Pdk4* mRNA by RT–qPCR. As shown in Fig4A, at 65 days of age SOD1^G86R^ mice had a 2.2-fold increase in *Pdk4* mRNA levels in TA when compared to WT mice (Fig4A). At the end stage of disease, *Pdk4* mRNA expression was 8.9-fold higher in SOD1^G86R^ mice when compared to age-matched WT littermates. Noticeably, in the *soleus* muscle of SOD1^G86R^ mice, *Pdk4* expression was comparable to that of WT mice at 65 and 105 days of age (Supplementary Fig S3). By contrast, *Pdk2* transcript levels were similar between SOD1^G86R^ mice and WT littermates in TA and in *soleus* at 65 days of age, but decreased by 2- and 0.3-fold in these respective muscles at 105 days of age (Fig4A and Supplementary Fig S3). In SOD1^G93A^ mice (Supplementary Fig S2C and D), *Pdk4* mRNA levels were transiently increased by 50% at onset when mice develop signs of hindlimb weakness, prior to returning to control levels at end stage, while *Pdk2* transcript levels were decreased by 50% from onset to end stage when compared to WT littermates. In muscle biopsies of definite ALS patients (Supplementary Fig S4), *Pdk4* mRNA levels were increased 3-fold while *Pdk2* mRNA levels remained unchanged when compared to controls.

Relative mRNA levels of *Pdk4* and *Pdk2* evaluated by qPCR at the indicated ages in *tibialis anterior* of WT and SOD1^G86R^ mice. Graphs represent mean fold change ± from age-matched WT. *Pdk4*: **P-values versus WT: 0.014 at 65 days and 0.018 at 105 days; *Pdk2*: ***P-value versus WT < 0.0001 at 105 days. *n* = 7 and 8 for WT and SOD1^G86R^, respectively, at 65 days; *n* = 5/genotype at 105 days, two-way ANOVA followed by Fisher's LSD *post hoc* test.

Relative mRNA levels of genes involved in lipid handling evaluated by qPCR at the indicated ages in *tibialis anterior* of WT and SOD1^G86R^ mice. Graphs represent mean fold change ± SEM from age-matched WT. *P*-values versus WT: *Lpl* **P =* *0.0009, *Cd36* *P =* *0.051, *Acsf2* ***P <* *0.0001 at 65 days, **P =* *0.0059 at 105 days and *Cpt-1β* **P =* *0.01 at 65 days and *P =* *0.02 at 105 days (*n* = 6 and 7 for WT and SOD1^G86R^, respectively, at 65 days; *n* = 10 and 8 for WT and SOD1^G86R^, respectively, at 105 days, two-way ANOVA followed by Fisher's LSD *post hoc* test).

Relative levels of (left) ATP and (right) NADH and NAD^+^ measured in total *tibialis anterior* homogenates of WT and SOD1^G86R^ mice. Left: Graphs represent mean fold change ± SEM in ATP from age-matched WT. **P =* *0.002 (*n* = 9 and 12 for WT and SOD1^G86R^, respectively, at 65 days; *n* = 10/genotype at 105 days, two-way ANOVA followed by Fisher's LSD *post hoc* test). Right: The amounts of NADH and NAD^+^ relative to protein content in whole *tibialis anterior* homogenates are represented as mean ± SEM. *P =* *0.027 and **P =* *0.001 (*n* = 5, Student's *t*-test).

PDK4 expression is stimulated by various parameters including skeletal muscle denervation, increased fatty acid (FA) use through β-oxidation (Jeong *et al*, 2012), and transcription factors peroxisome proliferator-activated receptors β/δ (PPARβ/δ) and forkhead Box O1A (FOXO1).

### NMJ dysfunction could participate in *Pdk4* up-regulation

To determine whether denervation could be responsible for the induction of *Pdk4* expression seen in ALS mice and patients, we used two complementary paradigms: a mild sciatic nerve crush that induces a transient denervation, and a chronic denervation obtained by sciatic nerve axotomy in WT mice. After sciatic nerve crush, mice were observed for paralysis of the ipsilateral hindlimb, sacrificed at various times (3 h to 8 weeks post-crush) and gene expression levels were measured in TA ipsilateral and contralateral to the site of nerve crush. Supplementary Fig S5A shows the rapid loss of the innervated NMJs starting from 1 day post-crush (DPC) to 5 DPC and the progressive reinnervation already visible after 1 week post-crush (WPC). As shown in Supplementary Fig S5B, the expression of AChRα started to increase (2-fold induction) at 1 DPC, reached a plateau between 3 DPC and 1 WPC with a 40- to 60-fold induction, and then returned to control level by weeks 4 and 8. *Pdk4* mRNA levels were increased by 50% during the early phase of synaptic destabilization with no significant changes on *Pdk2* mRNA levels (Supplementary Fig S5B). By contrast, *Pparβ/δ* and *Foxo1* (Supplementary Fig S5B) expression were rapidly and transiently stimulated by 2-fold. After sciatic nerve axotomy (Supplementary Fig S6), *AChRα*, *Pdk4*, *Pparβ/δ,* and *Foxo1* were still induced after 2 weeks of chronic denervation in both TA and *soleus* muscle while *Pdk2* mRNA levels were decreased. Collectively, these results show that NMJ destabilization and denervation could underlie *Pdk4* induction in TA muscle observed in SOD1^G86R^ mice.

### Pdk4 up-regulation is associated with an increase of the lipid handling pathway

Hence, we sought to better understand the metabolic significance of *Pdk4* up-regulation through RT–qPCR analyses of genes involved in lipid handling. When compared to WT littermates, 65-day-old SOD1^G86R^ mice had a significant increase in the expression of genes encoding lipoprotein lipase (*Lpl*, 81.2%), Cd36 (18.7%), acylCoA synthetase (*Acsf2*, 50%), and carnitine palmitoyl transferase 1B (*Cpt-1β*, 70%) (Fig4B). At the end stage of disease, the expression of *Lpl* and *Cd36* in SOD1^G86R^ mice was comparable to that of WT littermates, while *Acsf2* was decreased by 40% and *Cpt-1β* was increased by 45% (Fig4B). In SOD1^G93A^ mice (Supplementary Fig S2E and F), *Cpt-1β* and *Lpl* were decreased at the onset stage of disease and stayed below control levels until disease end stage. Only at this latter stage were *Pparβ/δ* and *Foxo1* mRNA levels significantly repressed or increased (Supplementary Fig S2G and H).

**Figure 5:**
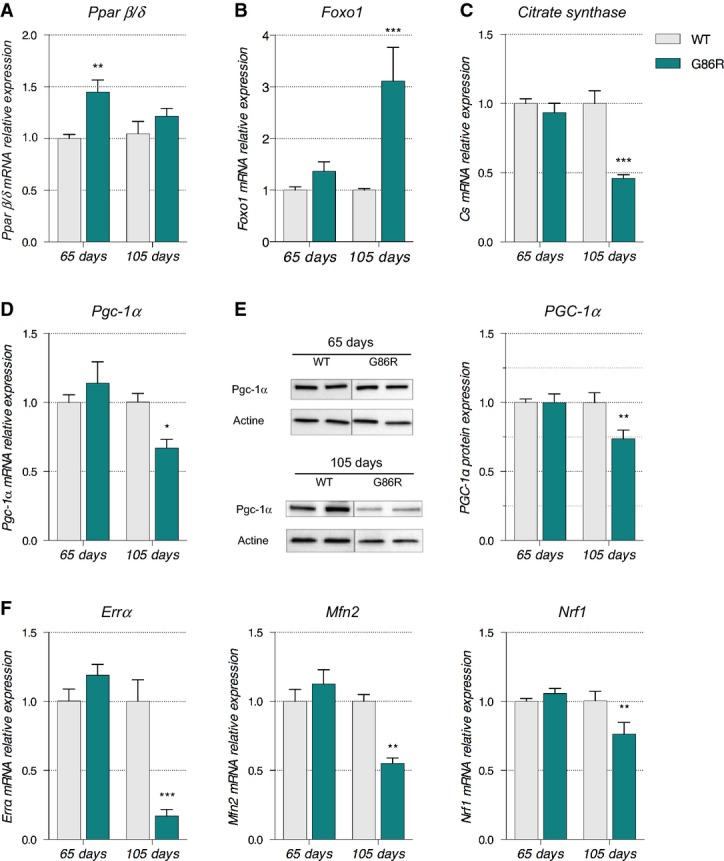
Transcription factors, PGC-1α, and citrate synthase are differentially regulated in SOD1^G86R^ mice

Increased expression of genes encoding an enzyme that hydrolyses triglycerides to facilitate the uptake of free fatty acids (LPL) (Mead *et al*, 2002), a membrane translocase that promotes FA entry into the cell (CD36), an enzyme that catalyses the conversion of FA to AcylCoA (ACSF2), and the rate-limiting enzyme for β-oxidation responsible for the FA transfer into the mitochondria (CPT-1B) (McGarry *et al*, 1983) in asymptomatic SOD1^G86R^ mice suggests that fatty acids intake is increased into glycolytic muscle tissue when ALS occurs. Taken together with the increased expression of *Pdk4*, our data indicate that higher levels of β-oxidation exist in glycolytic muscle prior to denervation. Given that adenosine triphosphate (ATP) and reduced nicotinamide adenine dinucleotide (NADH) are two energetic substrates produced by β-oxidation and oxidative phosphorylation in the mitochondria that are known to stimulate PDK activity (for review, see Harris *et al*, 2002), we measured the variation in these molecules in the TA of SOD1^G86R^ mice and WT littermates at 65 and 105 days of age. We evidenced a significant increase in ATP and NADH levels (1.37- and 1.49-fold change, respectively) in 65-day-old SOD1^G86R^ mice compared to WT while NAD^+^ levels were comparable (Fig4C). Interestingly, ATP and NADH levels were similar between SOD1^G86R^ mice and WT littermates at 105 days of age while NAD^+^ levels were increased 2.6-fold. Finally, mRNA levels of *Pparβ/δ* and *Foxo1* were increased in 65-day-old SOD1^G86R^ mice (Fig5A and B). Further, mRNA levels of *citrate synthase*, the first enzyme of the Krebs cycle, were comparable to WT at 65 days of age but significantly reduced at end stage (Fig5C). These data, together with the inhibition of glycolysis described in the previous section, clearly demonstrate that an early change in fuel preference from glucose toward lipids in glycolytic muscle fibers occurs before denervation in SOD1^G86R^ mice.

A–D Relative mRNA levels of (A) *Pparß/δ*, (B) *Foxo1*, (C) *citrate synthase*, and (D) *Pgc-1α* were evaluated by qPCR at the indicated ages in *tibialis anterior* of WT and SOD1^G86R^ mice. Graphs represent mean fold change ± SEM from age-matched WT. *P*-values versus WT: *Pparβ/δ* **P = 0.0016 at 65 days; *Foxo1* ***P =* *0.0001 at 105 days, *citrate synthase* ***P <* *0.0001 at 105 days, *Pgc-1α* *P = 0.0104 at 105 days (*n* = 7 and 8 for WT and SOD1^G86R^, respectively, at 65 days; *n* = 5/genotype at 105 days, two-way ANOVA followed by Fisher's LSD *post hoc* test).

E Representative Western blot of PGC-1α and respective actin that was used for normalization. PGC-1α protein levels in *tibialis anterior* muscle tissue are represented as mean fold change ± SEM from age-matched WT. **P-values versus WT: 0.02 (*n* = 6/genotype at 65 days; *n* = 5/genotype at 105 days, two-way ANOVA followed by Fisher's LSD *post hoc* test).

F Relative mRNA levels of *ERRα*, *Mfn2*, and *Nrf1* were evaluated by qPCR at the indicated ages in *tibialis anterior* of WT and SOD1^G86R^ mice. Graphs represent mean fold change ± SEM from age-matched WT. *P*-values versus WT: *ERRα* ***P <* *0.0001, *Mfn2* **P =* *0.002 and *Nrf1* **P =* *0.009 (*n* = 6–7 and 7–8 for WT and SOD1^G86R^, respectively, at 65 days; *n* = 5 and 6 for WT and SOD1^G86R^, respectively, at 105 days, two-way ANOVA followed by Fisher's LSD *post hoc* test).

### The metabolic switch in glycolytic muscle occurs independent of PGC-1α

**Figure 6:**
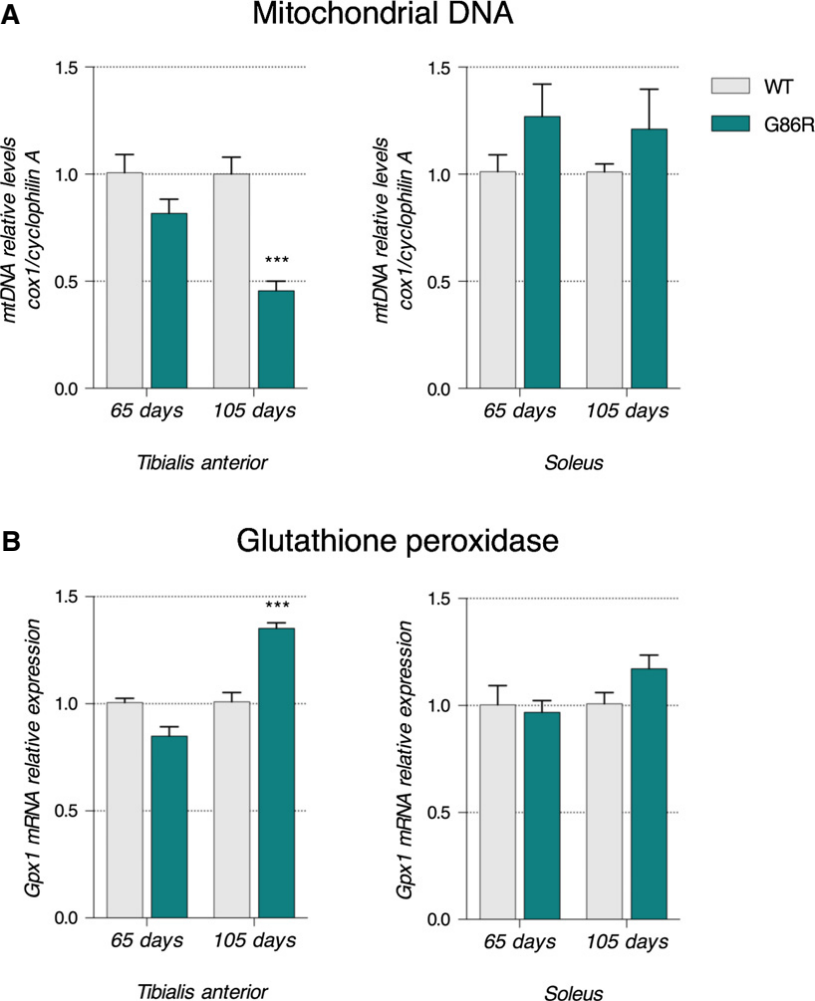
Altered mitochondrial function in glycolytic muscle of SOD1^G86R^ mice

The processes that promote the mobilization and use of fatty acids for ATP synthesis during moderate exercise are subjected to multiple regulatory steps. Typically, increased oxidative capacity and lipid use in muscle is accompanied by a switch in muscle fiber subtype, a phenomenon that is facilitated by peroxisome proliferator-activated receptor gamma co-activator 1-alpha (PGC-1α), the master regulator of mitochondrial biogenesis and function (Wu *et al*, 1999; Lin *et al*, 2002). We thus analyzed the expression of PGC-1α in the TA of SOD1^G86R^ mice. At 65 days of age, *Pgc-1α* mRNA and protein levels were similar between SOD1^G86R^ and WT mice (Fig5D and E), whereas they were significantly decreased at 105 days of age in SOD1^G86R^ mice. Importantly, at the end stage of the disease the decrease in PGC-1α protein levels correlated with a decrease in the mRNA expression of the main target genes of PGC-1α pathway, estrogen-related receptor α (*ERRα*), mitofusin 2 (*Mfn2*), and nuclear respiratory factor 1 (*Nrf1*) (Puigserver, 2005; Villena & Kralli, 2008) (Fig5E). *ERRα* mRNA expression was reduced by more than 5-fold, *Mfn2* mRNA levels were decreased by 2-fold, and *Nrf1* was decreased by 30% in SOD1^G86R^ mice when compared to WT animals at 105 days (Fig5F). In addition, mitochondrial DNA quantification indicated a decreased number of mitochondria in TA of SOD1^G86R^ mice toward the end stage of disease (Fig6A). Interestingly, this decrease in mitochondrial DNA was not observed in the *soleus* muscle. These data suggest that increased activation of the lipid oxidation pathways in skeletal muscle of SOD1^G86R^ mice occurs independently of PGC-1α and that this metabolic change does not have the support of the mitochondrial machinery of the cell. Presumably, this would have deleterious effects due to accumulation of β-oxidation by-products and ROS (Bonnard *et al*, 2008). Indeed, glutathione peroxidase 1 (*GPX1*), an important indicator of oxidative stress, was increased in the TA but not the *soleus* of SOD1^G86R^ mice at end stage of disease (Fig6B).

Mitochondrial DNA (mtDNA) was quantified using qPCR. Relative mtDNA levels are expressed as the ratio between mitochondrial-encoded gene *Cox1* and the nuclear-encoded gene *cyclophilin A*. Graphs represent mean fold change ± SEM from age-matched WT for *tibialis anterior* (left panel) and *soleus* (right panel) of WT and SOD1^G86R^ mice. ***P <* *0.0001 (*n* = 5 and 4 for WT and SOD1^G86R^, respectively, at 65 days; *n* = 6/genotype at 105 days, two-way ANOVA followed by Fisher's LSD *post hoc* test).

Relative mRNA levels of *Gpx1* were measured by qPCR at the indicated ages in *tibialis anterior* (left panel) and *soleus* (right panel) of WT and SOD1^G86R^ mice. Graphs represent mean fold change ± SEM from age-matched WT. ***P <* *0.0001 (*n* = 7 and 8 for WT and SOD1^G86R^, respectively, at 65 days; *n* = 9 and 8 for WT and SOD1^G86R^, respectively, at 105 days, two-way ANOVA followed by Fisher's LSD *post hoc* test).

### Restoring metabolic equilibrium facilitates weight gain and delays the onset of motor symptoms and denervation in SOD1^G86R^ mice

In order to ascertain whether decreased glycolytic capacity in SOD1^G86R^ mice significantly impacts weight loss (due to the mobilization of fat) and muscle function and pathology that is commonly observed in ALS (Dupuis *et al*, 2011), mice were treated with dichloroacetate (DCA), a halogenated organic acid that inhibits the activity of PDK and facilitates the entry of pyruvate into the Krebs cycle and the oxidation of glucose. By inhibiting PDK and preventing PDH phosphorylation, we aimed to force metabolism toward glucose oxidation (Abdel-Aleem, 1996), thereby reversing the metabolic alterations observed in SOD1^G86R^ mice, preventing weight loss, reducing the toxic effects of lipids on mitochondria, and delaying denervation in glycolytic muscle fibers. We administered DCA daily in drinking water from 60 to 95 days of age. At the beginning of treatment (T_0_), SOD1^G86R^ mice in both treated (DCA) and non-treated (CT) groups were significantly leaner that WT mice (Supplementary Fig S7A). Over five weeks of treatment, SOD1^G86R^ mice that received drinking water (CT) maintained their initial weight. By contrast, SOD1^G86R^ mice receiving DCA increased their initial weight by 10.8%, an increase that was similar to that observed in CT WT mice over the same treatment period (Supplementary Fig S7A and B). DCA had no effect on the weight gain of WT animals (Supplementary Fig S7A and B).

**Figure 7:**
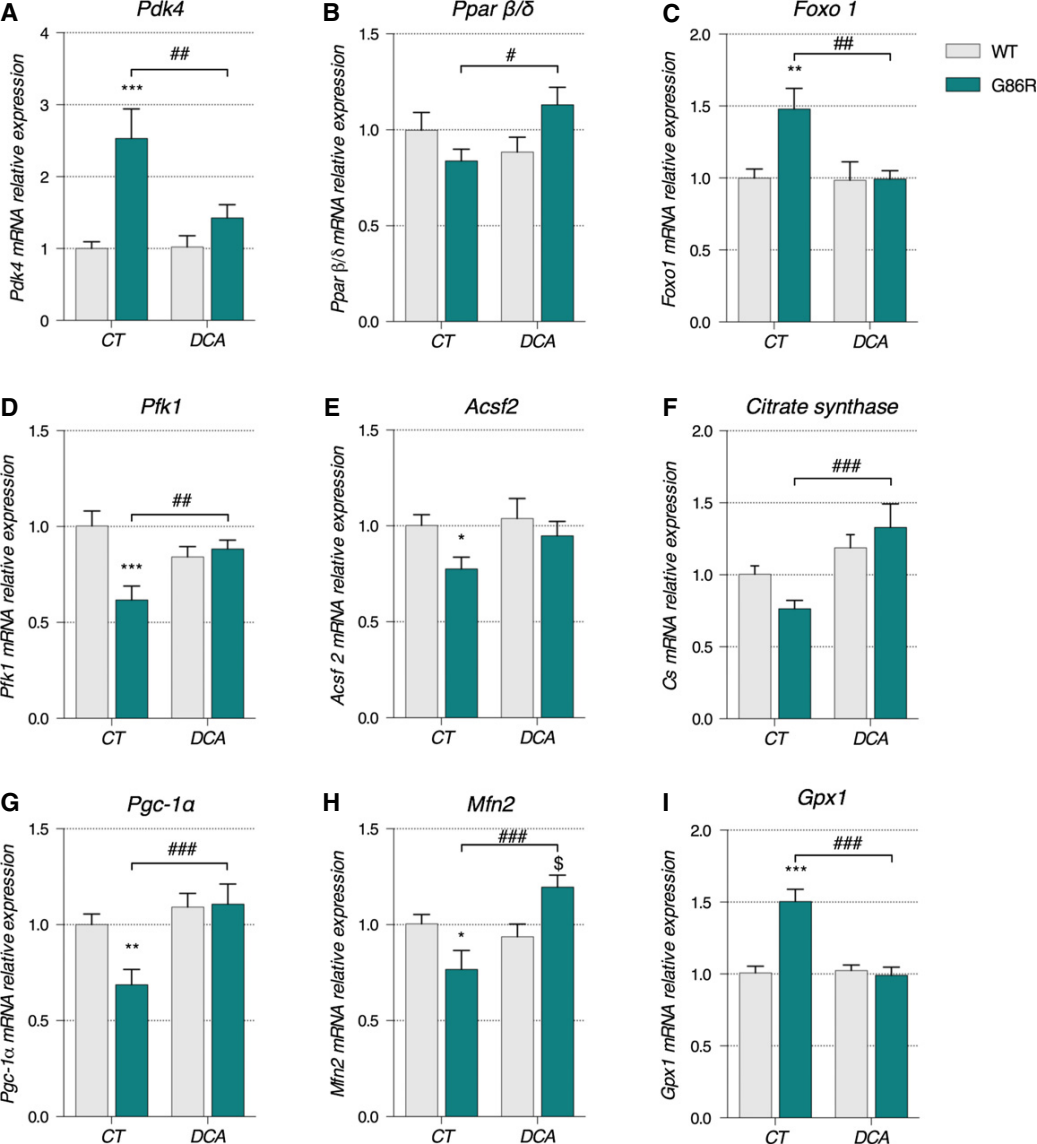
DCA treatment had beneficial effects on metabolism and mitochondrial function of SOD1^G86R^ mice

Daily administration of DCA was able to reverse the changes in *Pdk4*, *Pparβ/δ*, and *Foxo1* mRNA expression in SOD1^G86R^ mice (DCA) when compared to non-DCA-treated SOD1^G86R^ mice (CT, Fig7A–C). At the cessation of treatment, *Pfk1*, *Acsf2,* and *citrate synthase* mRNA levels in DCA-treated SOD1^G86R^ mice were comparable to DCA-treated WT mice (Fig7D–F), indicating successful restoration of glycolysis and activation of the Krebs cycle. Importantly, CT SOD1^G86R^ mice presented with significantly reduced *Pfk1* and *citrate synthase* expression when compared to DCA-treated SOD1^G86R^ mice and/or CT WT mice. DCA treatment was also able to preserve the expression of *Pgc-1α* and its target gene *Mfn2* in DCA-treated SOD1^G86R^ mice at levels comparable to that seen in CT WT and DCA-treated WT mice (Fig7G and H). The increase in the expression of genes involved in mitochondrial biogenesis was accompanied by a decrease in the expression of the *Gpx1* (Fig7I). Notably, and in support of our results above, 95-day-old CT SOD1^G86R^ mice presented with a significant decrease in the expression of *Pgc-1α* and *Mfn2* mRNA and an increase in the expression of *Gpx1* mRNA (Fig7G–I). Collectively, these data suggest that DCA is able to restore glycolytic function while decreasing oxidative stress in glycolytic muscle of SOD1^G86R^ mice.

A–I Relative mRNA levels of (A) *Pdk4*, (B) *Pparβ/δ*, (C) *Foxo1*, (D) *Pfk1*, (E) *Acsf2,* (F) *citrate synthase*, (G) *PGC-1α*, (H) *Mfn2,* and (I) *Gpx1* were evaluated by qPCR in *tibialis anterior* of control (CT) or DCA-treated (DCA) WT and SOD1^G86R^ mice. Graphs represent mean fold change ± SEM from CT WT group. *P*-values versus WT: *Pdk4* ***P =* *0.002 and ^##^*P *=* *0.0039, *Pparβ/δ* ^#^*P *=* *0.0178, *Foxo1* **P =* *0.0033 and ^##^*P *=* *0.0038, *Pfk1* ***P =* *0.0002 and ^##^*P *=* *0.0080, *Acsf2* *P =* *0.0437, *citrate synthase* ^###^*P *=* *0.0004, *Pgc-1α* **P =* *0.0084 and ^###^*P *=* *0.0009, *Mfn2* *P =* *0.0272, ^$^*P *=* *0.0145 and ^###^*P *=* *0.0002 and *Gpx1* ***P <* *0.0001 and ^###^*P *<* *0.0001 (*n* = 9/genotype in CT groups, *n* = 9 and 8 for WT and SOD1^G86R^, respectively, in DCA group, two-way ANOVA followed by Fisher's LSD *post hoc* test).

**Figure 8:**
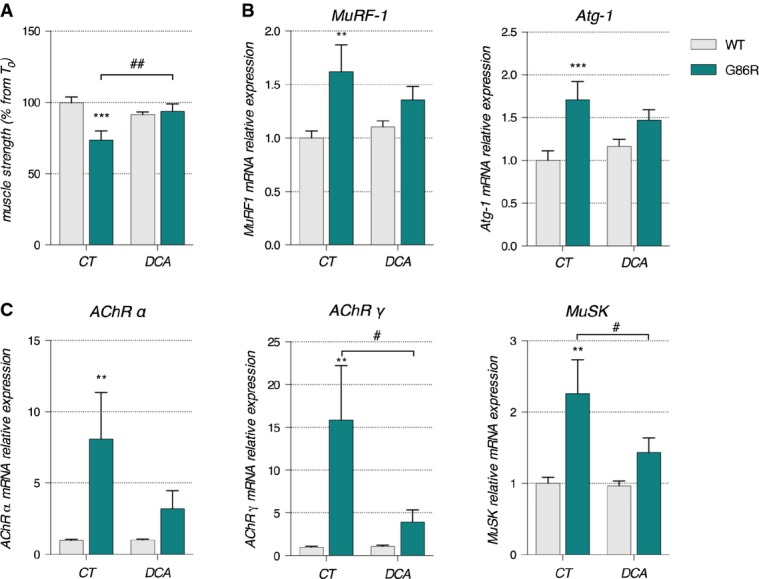
DCA treatment had protective effects on muscle strength and prevented the expression denervation markers

At a functional level, DCA was able to almost completely preserve muscle strength. While CT SOD1^G86R^ mice lost 30% of their grip strength, DCA-treated SOD1^G86R^ mice only lost 8% of their grip strength when compared to initial grip force at the initiation of treatment (Fig8A). This increased grip strength was associated with an increase in both oxidative and glycolytic muscle fiber size in TA (Supplementary Fig S7D and E). Analysis of mRNA expression of muscle atrophy markers showed a more modest improvement (Fig8B). *Murf-1* mRNA expression was reduced from a 1.6-fold increase in the non-treated CT SOD1^G86R^ mice to 1.3-fold increase in DCA-treated SOD1^G86R^ mice. Similarly, *Atg-1* mRNA expression was decreased from a 1.7-fold increase in CT SOD1^G86R^ mice to a 1.4-fold increase in DCA-treated SOD1^G86R^ mice. DCA treatment also prevented the increase in the expression of the denervation markers (Fig8C). The increase in expression of *AChRα* mRNA in DCA-treated SOD1^G86R^ mice was only 3.2-fold as compared to an 8.8-fold increase in CT SOD1^G86R^ mice. *AChRγ* mRNA was increased by 3.9-fold in DCA-treated SOD1^G86R^ mice as compared to a 15.87-fold increase measured in CT SOD1^G86R^ mice. Lastly, *MuSK* gene expression was also lower following treatment with DCA, decreasing from a 2-fold increase in CT SOD1^G86R^ mice to a 1.3-fold increase in DCA-treated SOD1^G86R^ mice. WT mice treated with DCA did not present with any modifications of these atrophy or denervation markers. Altogether, these results demonstrate that by facilitating the equilibrium between glucose and lipid oxidation through the administration of DCA, we are able to promote weight gain, restore mitochondrial gene expression, improve muscle strength, and decrease the expression of denervation markers in SOD1^G86R^ mice.

Grip strength is represented as mean of percent from T_0_ for each experimental group ± SEM. ^##^*P *=* *0.0045 and ***P =* *0.0003 (*n* = 9/genotype in CT groups, *n* = 9 and 8 for WT and SOD1^G86R^, respectively, in DCA group, two-way ANOVA followed by Fisher's LSD *post hoc* test).

Relative mRNA levels of muscular atrophy markers *Murf1* and *Atg-1* were measured by qPCR in *tibialis anterior* of control (CT) or DCA-treated (DCA) WT and SOD1^G86R^ mice. Graphs represent mean fold change ± SEM from CT WT group. **P =* *0.0079 and ***P =* *0.0018 (*n* = 9/genotype in CT groups, *n* = 9 and 8 for WT and SOD1^G86R^, respectively, in DCA group, two-way ANOVA followed by Fisher's LSD *post hoc* test).

Relative mRNA levels of denervation markers *AChRα*, *AChRγ*, and *MuSK* were measured by qPCR in *tibialis anterior* of control (CT) or DCA-treated (DCA) WT and SOD1^G86R^ mice. Graphs represent mean fold change ± SEM from CT WT group. **P =* *0.015 for *AChRα*, ^#^*P *=* *0.028 and **P =* *0.074 for *AChRγ*, ^#^*P *=* *0.042 and **P =* *0.0029 for *MuSK* (*n* = 9/genotype in CT groups, *n* = 9 and 8 for WT and SOD1^G86R^, respectively, in DCA group, two-way ANOVA followed by Fisher's LSD *post hoc* test).

## Discussion

Here, we provide evidence of a change in the metabolic capacity of glycolytic muscle in SOD1^G86R^ mice. A switch from glucose metabolism to lipid metabolism occurs early in the disease process and prior to any detectable motor and clinical (EMG alteration) symptoms. By improving glycolytic capacity in SOD1^G86R^ mice through administration of DCA, we promoted a delay in the onset of motor symptoms and amelioration of muscle denervation and atrophy. Thus, we demonstrate for the first time that an early switch to lipid metabolism in skeletal muscle may underlie early pathological mechanisms associated with NMJ destabilization in ALS.

The decreased capacity for SOD1^G86R^ mice to endure acute physical exercise that solicits anaerobic metabolism in muscle occurred before any measurable muscle weakness or overt denervation (Figs1 and 2). This low resistance to intense exercise contrasted their enhanced endurance capacity during acute aerobic exercise. This observation suggests that SOD1^G86R^ mice acquire new properties in skeletal muscle that enhance global aerobic capacity and promote endurance ability. It is plausible that altered glucose and insulin responses in SOD1^G86R^ mice (Supplementary Fig S1) might underlie this change in exercise capacity since insulin plays a critical role in driving glucose uptake into skeletal muscle for use as an energy substrate. Indeed, high incidences of glucose intolerance have been observed in patients diagnosed with sporadic ALS (Pradat *et al*, 2010). However, as basal glucose uptake in extensor digitorum longus (EDL: another glycolytic muscle) of the SOD1^G93A^ mouse model of ALS is normal before overt denervation (Smittkamp *et al*, 2014), it would seem unlikely that altered insulin sensitivity underlies the metabolic changes observed in this study.

Endurance exercise is also supported by slow-twitch oxidative type I fibers while intense exercise requires movements involving strength and speed that are generated by fast-twitch glycolytic type IIb fibers (Bassel-Duby & Olson, 2006). Given that a switch in fiber type from glycolytic to oxidative fibers has been described in ALS patient muscle biopsies (Telerman-Toppet & Coërs, 1978), and in the SOD1^G93A^ mouse model of ALS (Atkin *et al*, 2005; Hegedus *et al*, 2008; Deforges *et al*, 2009), the observed alteration in exercise capacity in SOD1^G86R^ mice might also reflect a switch of muscle fiber type. In line with this, enhanced aerobic capacity is typically observed after endurance training, and this occurs concurrent with measurable changes in fiber type composition (Pette & Staron, 2001).

**Figure 9:**
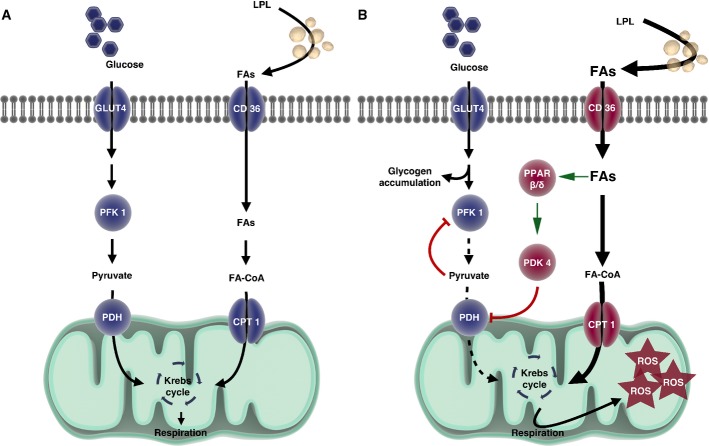
Schematic representation of the described metabolic imbalance in glycolytic muscle fibers of SOD1^G86R^ mice

PFK1 is the rate-limiting enzyme of the glycolysis, and an increase in glycogen synthase activity and glycogen accumulation in skeletal muscle is characteristic of a muscle that is subjected to endurance training (Vestergaard, 1999). Thus, the increase in endurance capacity in SOD1^G86R^ mice mirrors a profound alteration of fuel preference in muscle fibers. In accordance with this, we observed a progressive decrease of PFK1 activity, and an increase in glycogen synthase activity and glycogen accumulation in skeletal muscle. The initial decrease in PFK1 activity in SOD1^G86R^ mice may be an adaptive response to the progressive accumulation of pyruvate and increased FA uptake, two well-known potent inhibitors of PFK1 (Massao Hirabara *et al*, 2003). Furthermore, as the entry of glucose into muscle fibers in ALS mice is not affected early in disease (Dupuis *et al*, 2004; Smittkamp *et al*, 2014), our data suggest that glucose is rerouted toward glycogen stores rather than being used as an immediate energy source. By the end stage of disease, reduced activities of PFK1 and glycogen synthase, together with an increase of glycogen stores and reduced levels of pyruvate, demonstrate inhibition of the glycolytic pathway. Collectively, these observations suggest that glycolytic muscle is no longer able to mobilize glycogen stores to produce energy and that the inhibition of glycolysis appears to originate downstream of PFK1 (Fig9).

Under normal conditions in WT mice, both glucose and lipids enter the cell and are used as cellular fuels. In this situation, both pathways are used and are driven by PDK4 according to needs and fuel availability.

In the case of ALS, metabolic flexibility is lost because of chronically increased PDK4 and subsequent inhibition of PDH and PFK1 leading to glycogen accumulation. Increased lipid use through β-oxidation that can only function under aerobic conditions may underlie the greater endurance capacity in SOD1^G86R^ mice. With time, the cell produces more reactive oxygen species, thereby inducing cellular damage. Data information: CD36, CD36 antigen/fatty acid translocase, CPT1, carnitine palmitoyl transferase 1, FAs, fatty acids, FA-CoA, acyl-fatty acids, GLUT4, glucose transporter 4, LPL, lipoprotein lipase, PDH, pyruvate dehydrogenase complex, PDK4, pyruvate dehydrogenase kinase 4, PFK1, phosphofructokinase 1, ROS, reactive oxygen species. Drawing was performed with the website Somersault1824 (http://www.somersault1824.com).

In contrast to inhibition of glycolysis in SOD1^G86R^ mice, the lipid pathway was stimulated at 65 days of age and remained functional until the end stage of disease (Fig4B and C). It is known that increased endurance should correlate with increased release of fatty acids from lipid stores, and enhanced uptake by muscle tissue (Talanian *et al*, 2010). Previous observations of decreased fat pad mass and respiratory quotient at an early stage in SOD1^G86R^ mice (Dupuis *et al*, 2004) and increased lipid clearance in these mice (Fergani *et al*, 2007) are indicative of increased lipid use. Moreover, in patients diagnosed with sporadic ALS, Pradat and colleagues reported increased circulating free fatty acids (FFAs) (Pradat *et al*, 2010). Altogether, these observations indicate that muscle tissue of SOD1^G86R^ is the potential site of abnormal lipid consumption. The question raised from these observations was whether the more ‘efficient’ oxidation during exercise is due to a given group of muscles that work more efficiently or whether other groups of muscles are becoming oxidative to participate in the exercise task. The latter appears to occur since fiber type switching toward more oxidative metabolism (Deforges *et al*, 2009), and increased expression of genes encoding enzymes involved in lipid metabolism (Dupuis *et al*, 2004; Fergani *et al*, 2007) is observed at later, symptomatic stages of disease in ALS mouse models. In line with this, we show an early increase in the gene expression of important components in lipid mobilization and uptake (*Lpl*, *Cd36*, *ACSF2*, and *Cpt-1B*, Fig4B), and increased β-oxidation by-products (ATP and NADH at 65 days) that activate PDK4 and inhibit PDH activity (Denton *et al*, 1975) in fast-twitch glycolytic muscle.

PDK4 appears to play a pivotal role in the switch in fuel preference by orchestrating substrate competition in SOD1^G86R^ glycolytic muscle fibers. In skeletal muscle, PDK4 is the most highly expressed pyruvate dehydrogenase kinase isoform and is considered to be a critical regulator of pyruvate dehydrogenase (PDH) activity (Bowker-Kinley *et al*, 1998). In this study, we show that *Pdk4* expression is induced not only in two mutant SOD1 mouse models but also in sporadic ALS patients (Fig1, Supplementary Figs S2 and S4). These data show that the induction of *Pdk4* expression is not specific to SOD1 mutations but is rather an ALS-specific phenomenon. By phosphorylating PDH, PDK4 completely inhibits the entry of pyruvate into the Krebs cycle, thus hampering glucose oxidation (for review, see Holness & Sugden, 2003). PDH and PDK4 are known to act as metabolic homeostat in response to energy imbalance. Indeed, PDK4 transcription is increased during prolonged exercise, after both short-term high-intensity and prolonged low-intensity exercise or during fasting, situations that represent metabolic states where the whole body glucose availability is in deficit (Spriet *et al*, 2004; Watt *et al*, 2004; Jeong *et al*, 2012). *Pdk4* expression is controlled directly or indirectly by various transcription factors/transcriptional coactivators or stimuli, including PPARβ/δ, FOXO1, PGC-1α, ERRα, or FAs. In C2C12 mouse myoblasts, nutrient deprivation induces *Pdk4* gene expression (Furuyama *et al*, 2003). In rat muscle tissue, triglyceride infusion leads to increased plasma FA and increased expression of *Pdk4* mRNA (Kim *et al*, 2006). Moreover, it has been shown that in response to increased entry of FA in skeletal muscle or to fasting, induction of PPARβ/δ stimulates FOXO1 transcription, which in turn activates *Pdk4* expression (Nahlé *et al*, 2008). Here, we show that *Pdk4* activation might occur in response to the increase in *Pparβ/δ* and *Foxo1* mRNAs, rather than via the classical transcription factors PGC-1α/ERRα. If this were the case, then an increase of FA release due to the induction of *Lpl* and *Acsf2* would stimulate PDK4 expression via PPARβ/δ and FOXO1, which would in turn facilitate FA oxidation by conserving pyruvate for oxaloacetate formation to allow entry of acetyl-coA into the Krebs cycle (Fig1). In SOD1^G93A^ mice, although induction of *Pdk4* expression is detected at the onset (63–75 days old), the global metabolic profile of the mice differs from the one found in SOD1^G86R^ mice. It is highly likely that the observed differences are a result of profound mitochondrial alterations which have been described in the SOD1^G93A^ mice as early as post-natal day 30 (for review, see Vinsant *et al*, 2013a, b).

The question of the role of NMJ destabilization in the metabolic switch clearly remains open. We showed here that synaptic alteration/dysfunction obtained 24 h after sciatic nerve crush is sufficient to induce an early but transient increase in *Pdk4* mRNA, which then returns to initial levels despite a sustained increase in the levels of *AChRα* mRNA, an accepted marker of NMJ denervation. In the SOD1^G86R^, *Pdk4* mRNA is induced before any detectable sign of denervation by EMG and before the increased expression of the denervation makers used in this study. However, we cannot exclude a lack of sensitivity of our techniques. In the SOD1^G93A^ mice, denervation events have been detected at 30 days of age through a detailed study of the NMJ by electron microscopy (Vinsant *et al*, 2013a,b), and impaired neuromuscular transmission has been detected at 28–42 days of age (Rocha *et al*, 2013), long before the onset of motor symptoms. Here, we showed that, at the same period, *Pdk4* mRNA levels are comparable to those found in WT mice. Altogether, these data show that NMJ dysfunction or destabilization might participate to the *Pdk4* mRNA induction.

Another interesting aspect of the mechanism described here is the consequence of prolonged use of lipids as an energy substrate. Increased β-oxidation of fatty acids leads to the generation of lipid by-products that contribute to lipotoxicity and ROS production (Zhang *et al*, 2010; Aon *et al,* 2014). ROS accumulation in muscle tissue of SOD1^G86R^ mice is an early event in the course of the pathology that precedes overt signs of denervation (Halter *et al*, 2010). Here, we show an up-regulation of *Gpx1*, an indicator of ROS accumulation, in the TA but not in the *soleus* muscle of SOD^G86R^ mice in the advanced stages of the disease. It is expected that this chronic/long-term activation of lipid use would have toxic effects on mitochondria (Bonnard *et al*, 2008), which might explain the observed decrease of citrate synthase (Fig5C), the decrease in the expression of genes responsible for mitochondrial dynamics, and the reduction in the quantity of mtDNA specific to glycolytic muscle (Fig6). Indeed, rescuing muscle mitochondria in a mouse model for ALS through the overexpression of muscle-specific PGC-1α has been shown to prevent muscle atrophy and improve mitochondrial function (Da Cruz *et al*, 2012). Although PGC-1α is often considered as the main ‘fiber type regulator’ that contributes to muscle physiological plasticity under normal physiological conditions, PGC-1α is down-regulated with disease progression in our SOD1^G86R^ mice and can therefore not account for the induction of *Pdk4*. Alternatively, muscle-specific over-expression of PPARβ/δ in skeletal muscle results in a greater number of oxidative type 1 fibers (Luquet *et al*, 2003), and over-expression of constitutively active PPARβ/δ results in mitochondrial biogenesis and a shift from fast-twitch to slow-twitch fibers (Wang *et al*, 2004). Thus, the early switch of fuel preference in ALS mice could be driven by the increased expression of PPARβ/δ. Altogether, these findings show that metabolic imbalance in muscle fibers of SOD1^G86R^ mice is an early event. As glycolytic muscle fibers become progressively unable to use glucose as an energy substrate, they switch to lipid use to maintain a sufficient amount of energy supply. Intriguingly, this mechanism is specific to glycolytic muscle fibers as the deregulations observed in the TA differ from that seen in the oxidative *soleus* muscle.

Because PDK4 appears to be a key player in the switch in fuel preference by suppressing glucose utilization, we used the halogenated carboxylic acid DCA, a specific inhibitor of PDK, to restore glucose metabolism and muscle metabolic plasticity. Indeed, maintaining PDH in its unphosphorylated active form will re-activate PDH function and recover glucose metabolism by restoring glucose oxidation (Whitehouse & Randle, 1973). In a model of statin-induced muscle atrophy, DCA treatment has been shown to increase glucose oxidation in type IIb muscle fibers and reduce the expression of markers of protein degradation (Mallinson *et al*, 2012). This is in line with the results presented here. In this study, we demonstrate that DCA treatment exerts protective effects on muscle fibers in the SOD1^G86R^ mice by restoring the ability of glycolytic muscle to use glucose as fuel. When compared to non-DCA-treated SOD1^G86R^ mice, those supplemented with DCA show a decrease in the mRNA expression of *Pdk4*, *Foxo1*, and *Pparβ/δ* in parallel with an induction of *Pfk1* and *citrate synthase*. Moreover, DCA-treated SOD1^G86R^ mice also had decreased expression of denervation and atrophy markers when compared to untreated SOD1^G86R^ mice. At a functional level, this was reflected by a preservation of muscle strength and the presence of larger muscle fibers in DCA-treated SOD1^G86R^ mice compared to untreated SOD1^G86R^ mice. DCA treatment in SOD1^G86R^ mice also exerted beneficial metabolic effects in the whole organism by stimulating weight gain during disease development, which is similar to what has been observed after DCA treatment of statin-induced myopathy in mice (Mallinson *et al*, 2012). It is important to note that DCA treatment was able to restore some of the biochemical parameters that govern fuel use in SOD1^G86R^ mice (Fig7). We observed an up-regulation of mRNA expression of *Pgc-1α* and *Mfn2,* a PGC-1α target gene that is involved in mitochondrial dynamics. While DCA has not been shown to directly regulate the PGC-1α pathway, it has been shown to reverse the inhibition of PGC-1α and mitochondrial biogenesis that is induced in myotubes by chronic inhibition of glucose oxidation with pyruvate (Philp *et al*, 2010). The beneficial effects on mitochondrial metabolism following DCA treatment in SOD1^G86R^ mice were also reflected by comparable levels of *Gpx1* in DCA-treated SOD1^G86R^ mice and CT- and DCA-treated WT mice. Collectively, these data show that restoration of metabolic equilibrium in glycolytic muscle fibers is able to protect muscle mitochondria and prevent oxidative stress, while also preventing denervation and atrophy.

DCA has previously been used in the SOD1^G93A^ mouse model of ALS (Miquel *et al*, 2012). By limiting pyruvate turnover to lactate and facilitating the entry of pyruvate into the Krebs cycle, DCA reduced astrogliosis and motor neuron death (Miquel *et al*, 2012). This indicates that PDK activation may be a common mechanism in astrocytes and muscle. Interestingly, Miquel and colleagues also demonstrated that DCA-treated SOD1^G93A^ mice presented with a marked improvement in the structure of NMJs in the *extensor digitorum longus* (glycolytic muscle fiber) but not *soleus* (oxidative muscle fiber). Taken together with our data showing an effect of DCA in restoring glycolysis in muscle, one can conclude that DCA treatment exerts its protective effects through stabilizing the NMJ. However, based on the action of DCA on both muscle and astrocytes, we cannot conclude whether the normalization of glycolytic muscle metabolism is sufficient in itself to maintain the positive effect observed at the NMJ. However, our data provide convincing evidence that DCA treatment promotes the use of glucose as the preferred energy substrate (instead of lipids), thereby preventing denervation and maintaining function at NMJ.

In conclusion, our study provides compelling evidence to support a dramatic change in glycolytic muscle metabolism that specifically alters muscle metabolic plasticity in highly susceptible glycolytic fibers. Importantly, these metabolic alterations occur very early in the course of the disease. These data, in combination with our sciatic nerve crush and axotomy experiments, suggest that glycolytic defects might be due to additional events distinct from denervation. However, early changes of synaptic functionality might contribute to early stages of the metabolic switch, which in turn could contribute to further NMJ changes and a self-propagating process. Importantly, since altered metabolic plasticity might underlie the susceptibility of glycolytic fibers to denervation while limiting the capacity for muscle to become re-innervated, this would have detrimental effects on the rate of disease progression. By demonstrating the involvement of multiple cell types in ALS pathology and identifying a possible common metabolic thread between glycolytic muscle and astroglia in ALS pathogenesis, our work further exemplifies the complexity of ALS. Finally, our work has important therapeutic implications as we present evidence to suggest that by improving metabolic function in murine ALS animal model through DCA, it is possible to improve motor function, maintain muscular integrity, and delay denervation.

## Materials and Methods

### Ethics statement

All experiments performed with mice followed current European Union (Directive 2010/63/EU) and Australian regulations. They were approved by the regional ethics committee CREMEAS 35 under number AL/01/20/09/12 and by The University of Queensland Animal Ethics Committee (ethics number SBMS/562/12/MNDRIA). They were performed in accordance with national guidelines and in strict compliance with the recommendations in the Guide for the Care and Use of Laboratory Animals of the National Institutes of Health (USA) by authorized investigators.

For human biopsies, the study was approved and performed under the ethical guidelines issued by our institution (University Hospital of Strasbourg) for clinical studies. The diagnostic procedures were conducted according to the Strasbourg University Hospital Ethical Committee, and informed written consent was obtained from all patients.

### Muscle biopsy collection

We studied biopsies from the left deltoid muscle of eleven patients with sporadic ALS. According to the El Escorial criteria, all patients had definite ALS. The control group included seven patients subjected to a standard surgical diagnosis procedure without significant neurological history. All patients gave written informed consent before biopsy. Tissues were immediately frozen in liquid nitrogen and stored at −80°C until use.

### Animals

FVB/N males overexpressing murine *SOD1* with the G86R mutation or C57/Bl6 males overexpressing human *SOD1* with the G93A mutation [B6.Cg-Tg(SOD1-G93A)1Gur/J] were genotyped as described previously (Gurney *et al*, 1994; Ripps *et al*, 1995). Wild-type littermates were used as controls. SOD1^G93A^ mice were studied at predefined stages of disease progression (Ngo *et al*, 2012). Male Thy1-YFP mice on FVB background (Feng *et al*, 2000) were used for direct identification of presynaptic terminals after nerve crush. Mice were group-housed (2–5 per cage) in a temperature- and humidity-controlled environment at 23°C and under a 12-h light/dark cycle. Mice had access to water and regular A04 chow *ad libitum*. Mice with a body weight outside of the standard at the time of the selection were excluded from the experiments. Blinded investigators were employed to minimize the effect of subjective bias when assessing results.

#### Exercise paradigms

The week before starting the experiment, all animals were acclimated to the treadmill exercise (Treadmill Control, Letica, Spain) by running at 25 cm/s with a 5° inclination for 5 min for 3 days. Mice were tested at 65–66 days of age.

#### Intense anaerobic exercise

A maximal incremental test was carried out. The incline was set at + 10° and the speed at 40 cm/s and was maintained at this rate for 2 min. The speed was then increased by 3 cm/s every 90 s until exhaustion of the animal. The speed at which each mouse stopped running was considered to be the maximal running speed (*V*_max_).

#### Low-intensity endurance exercise

To determine maximal endurance capacity, a rectangular test was established. All mice ran at 80% of the *V*_max_ attempt during the maximal incremental test. The incline was set at + 10°, and after 2 min of acclimation at 40 cm/s, the speed was maintained at 80% of the *V*_max_ until exhaustion.

The criterion for exhaustion was a time of 5 s spent on the electrical grid without running. Blood samples from the tip of the tail were obtained immediately at the end of exercise to measure blood lactate using a lactate pro-LT device (Lactate Pro LT-1710, Arkray®, China).

### Muscle grip strength

Muscle grip strength was measured using a strength meter (Bioseb Grip test, Bioseb, France). Animals were placed over a metallic grid that they instinctively grab to stop the involuntary backward movement carried out by the manipulator until the pulling force overcomes their grip strength. Each animal was pulled over the entire length of the metallic grid until it lost its grip. Grip strength, expressed in Newton, was recorded over three trials for each animal in each test session. The maximal grip strength was obtained by averaging the three grip strength scores for each animal. All recordings were performed by the same blinded investigator to minimize variability in the experimental procedure.

### Electromyography

All recordings were performed with a standard EMG apparatus (Dantec, Les Ulis, France) in accordance with the guidelines of the American Association of Electrodiagnosis Medicine (AAEM). Mice were anesthetized with 100 mg/kg ketamine chlorhydrate and 5 mg/kg xylazine and maintained at 36°C over a thermostatic blanket. In order to minimize variability, all recordings were performed by the same blinded investigator. Recordings were monitored for 2 min with a concentric needle electrode (9013S0011, diameter 0.3 mm; Medtronic, USA) inserted into the *gastrocnemius* muscle. A monopolar needle electrode (9013R0312, diameter 0.3 mm; Medtronic) was inserted into the tail of the mouse to ground the system. Recordings showing voluntary activity were discarded. Spontaneous activity (mainly fibrillations) characteristic of muscle denervation was differentiated from voluntary activity (regular discharges that disappear with muscular relaxation) by visual and auditory inspection. Spontaneous activity with a peak-to-peak amplitude of at least 50 μV was considered to be significant.

### Glucose tolerance test and insulin tolerance test

65-day-old male transgenic and non-transgenic mice were fasted for 18 h (starting at 6 PM) prior to the glucose tolerance test (GTT) or fasted for 4 h (starting at 9 AM) prior to the insulin tolerance test (ITT). Blood glucose levels were evaluated using a commercial FreeStyle Papillon InsuLinx glucometer (Abbot, France). Blood was drawn from a small incision at the tip of the tail. For GTTs, initial blood glucose was measured prior to an IP injection of 2 g/kg body mass of glucose. For ITTs, insulin from bovine pancreas (Sigma-Aldrich, USA) was injected IP (0.5 U/kg body mass) after initial blood glucose was measured. Changes in blood glucose were followed for 120 min with measurements taken every 15 min for both GTTs and ITTs. For each mouse, blood glucose was expressed as the percentage of initial blood glucose concentration (T0).

### Sciatic nerve crush and axotomy

Axotomy of the sciatic nerve was performed on 8- to 9-week-old non-transgenic FVB/N male mice. Sciatic nerve crush was performed on 8- to 9-week-old non-transgenic or Thy1-YFP male mice on a FVB background. Mice were anesthetized by intraperitoneal injection of 100 mg/kg ketamine chlorhydrate and 5 mg/kg xylazine. The sciatic nerve was exposed at mid-thigh level and either crushed with a fine forceps for 30 s (mild nerve crush) or sectioned using microscissors (axotomy). For the sham animals, skin was opened and the sciatic nerve was exposed without additional manipulation. The skin incision was sutured, and mice were allowed to recover. Contralateral hindlimbs served as control. The efficiency of nerve crush was confirmed 24 h after injury by gait abnormalities and defects in paw flexion when the mouse was held by the tail. Mice were sacrificed by lethal injection of pentobarbital at different time points following the surgical intervention. Muscles were dissected, snap-frozen for biochemistry (FVB/N mice), or fixed with 4% buffered paraformaldehyde for 30 min for NMJ analysis (Thy1-YFP mice).

### NMJ analysis

Fixed muscles from Thy1-YFP mice were cut into bundles. Bundles were incubated 1 h with tetramethyl rhodamine-conjugated α-bungarotoxin (αBGT, 1 μg/ml in PBS; Sigma). Fluorescence staining was monitored with a laser-scanning microscope (confocal Leica SP5 Leica Microsystems CMS GmbH). Z-stacks of 2-μm optical sections were analyzed using ImageJ freeware (http://rsbweb.nih.gov/ij/).

### Dichloroacetate treatment

The pharmacological treatment consisted in daily administration of dichloroacetate (DCA; Sigma-Aldrich) in drinking water at a concentration corresponding to a daily dose of 500 mg/kg body mass as previously described (Miquel *et al*, 2012). The concentration was calculated based on a predicted water intake of 5 ml/day/animal (Bachmanov *et al,* 2002). For each genotype, male wild-type and SOD1^G86R^ littermate mice were randomly allocated into experimental groups. Mice with a body weight outside of the standard at the time of the selection were excluded. Water intake was carefully monitored throughout the duration of the experiment. Standard drinking water was used as a control (CT). Mouse weights were monitored weekly by an investigator who was blinded to mouse genotypes and treatment groups.

### Quantitative RT–PCR

Frozen muscle samples were homogenized in 1 ml/100 mg tissue TRIzol reagent (Invitrogen, USA) together with a stainless bead. Two 3-min homogenization cycles were performed in a TissueLyser (Qiagen, Germany) at 30 Hz. RNA was extracted using chloroform/isopropyl alcohol/ethanol and stored at −80°C. One microgram of total RNA was used to synthesize cDNA using Iscript reverse transcriptase (Bio-Rad, USA) and oligo-dT primer as specified by the manufacturer. Gene expression was measured using the 2× SYBR green SsoAdvanced reagent (Bio-Rad) according to the manufacturer's instructions on a Bio-Rad iCycler. PCR was performed under optimized conditions as follows: 95°C denaturation for 30 s, followed by 40 cycles of 10 s at 95°C and 30 s at 60°C. The extended list of primers (Eurogentec, Belgium) is provided in Supplementary Table S1. Relative expression was achieved by calculating the ratio between the cycle number (*C*_t_) at which signal crossed a threshold set within the logarithmic phase of the gene of interest and that of TBP housekeeping gene. *C*_t_ values were means of duplicates.

### Quantification of mitochondrial DNA

Snap-frozen tissue was digested in KTT buffer (Tris 10 mM pH 9, Triton X-100 0.1%, KCl 50 mM) with 2% proteinase K (Sigma-Aldrich) overnight at 54°C under constant agitation. Proteinase K was inactivated at 100°C for 5 min. RNA was degraded with 0.3 μg/μl RNase for 1 h at 37°C. Total DNA was extracted using a mix of Roti-Phenol reagent (Roth, USA), chloroform, and isoamyl alcohol (25/24/1). DNA was quantified using RT–qPCR (initial denaturation step at 98°C for 2 min, followed by 40 cycles of 10 s at 98°C and 20 s at 62°C). The relative mtDNA levels were calculated by normalizing the relative expression of mitochondrial Cox1 to the relative expression of the nuclear cyclophilin A. Primer sequences are provided in Supplementary Table S1.

### Western blotting

Snap-frozen muscle tissue was pulverized in a TissueLyser (Qiagen) for 2 × 20 s under liquid nitrogen using stainless steel beads. Tissue powder was homogenized in RIPA lysis buffer (50 mM Tris pH 7.4, 150 mM NaCl, 1 mM EDTA, 1% Triton X-100, 0.1% SDS, 0.5% sodium deoxycholate) at 1 ml/100 mg tissue containing 1:100 protease inhibitor cocktail (Calbiochem, USA) and phosphatase inhibitor cocktails 2 and 3 (Sigma-Aldrich, Germany). Determination of protein concentration was carried out using a BCA Assay Reagent Kit (UP95424 Uptima, France). Protein was denaturated by boiling, resolved by sodium dodecyl sulfate–polyacrylamide gel electrophoresis and transferred to 2-μm nitrocellulose membranes (Bio-Rad, France) using a semi-dry Transblot Turbo system (Bio-Rad, France). After using a chemiluminescent blocker (Millipore, France), membranes were probed with primary antibodies against Glycogen Synthase Rabbit mAb (Cell Signaling; Cat#3886; 1:500), Phospho-Glycogen Synthase Ser641 (Cell Signaling; Cat#3891; 1:500), PGC-1α (Millipore; Cat#AB3242, 1:1,000), and total actin (Sigma-Aldrich; Cat#A2103, 1:10,000). Primary antibodies were detected with anti-rabbit HRP (P.A.R.I.S; Cat#BI2407, 1:5,000). The protein bands were detected by chemiluminescence using ECL Lumina Forte (Millipore) and a chemiluminescence detector (Bio-Rad, France).

### Phosphofructokinase enzymatic activity

For biochemical analysis, mice were sacrificed and tissues were quickly dissected, frozen in liquid nitrogen, and stored at −80°C until use. All chemicals were purchased from Sigma-Aldrich. Frozen *tibialis anterior* muscles were pulverized with a mortar and pestle under liquid nitrogen. The powder was resuspended in five volumes (w/v) of extraction buffer containing 20 mM Tris–HCl pH 7.5, 0.15 M NaCl, and Protease inhibitor cocktail (Calbiochem, USA). Samples were homogenized by vortexing for 3 min prior to incubation for 10 min on roller at room temperature. After a final homogenization by vortexing for 2 min, samples underwent centrifugation for 15 min at 10,000 *g* at 4°C. The supernatant was used for the assay of PFK activity and determination of protein concentration as described above.

PFK activity was determined at 25°C in 96-well microplates in a total volume of 240 μl. An aliquot (10 μl) of tissue extract was added to 200 μl of reaction cocktail solution (50 mM Tris–HCl pH 8.0, 3 mM MgCl_2_, 3 mM DTT, 0.1 mM EGTA, 0.3 mM NADH, 2 mM fructose-6-phosphate, 10 IU aldolase, 80 IU triose phosphate isomerase, 14 IU glycerol-3-phosphodeshydrogenase, and 10 μl of distilled water). The reaction was started with the addition of 20 μl of 10-mM ATP. PFK activity was measured every minute over 20 min and calculated from the linear rate of change in absorbance at 340 nm (Tristar LB 941 apparatus, Berthold Technologies, Germany). Calculations were based on the extinction coefficient of 6.22 × 10^−3^ M for NADH and corrected by the volume of the sample added, to obtain nmol of NADH oxidized/min/μl of sample. As one mole of NADH corresponds to one mole of PFK, the activity of PFK was calculated for each sample relative to the protein concentration of each sample.

### Intermediary metabolites

All intermediary metabolites analyzed were extracted from fresh snap-frozen tissue. Quantification was performed using commercial colorimetric assays for pyruvate (K609-100; BioVision, USA), ATP (K354-100; BioVision), and NAD^+^ and NADH (ab65348; Abcam, UK). Samples were deproteinized using 10-kDa cutoff spin filters (ab93349; Abcam), and assays were performed according to manufacturer's instructions. The optical densities for all assays were obtained using a Tristar LB 941 multimode plate reader (Berthold Technologies, France), and raw concentration values were calculated as indicated by the manufacturer.

### Histochemistry and morphometry

Glycogen deposition in muscle biopsies was determined using the standard periodic acid-Schiff (PAS) stain. Transverse (10 μm) TA sections were obtained from snap-frozen tissue embedded in TissueTek O.C.T. compound (Sakura, Japan) on a cryostat (Leica CM 3050S, Germany) at −24°C. Freshly cut sections were briefly dried at 37°C for 30 min. Sections were then incubated for 10 min in periodic acid 1% (Roth, Germany), followed by 30 min in Schiff's reagent (Roth, Germany) prior to a 5-min counterstain in Mayer's solution (Roth, Germany). After thorough washing, slides were dehydrated with successive alcohol–toluene baths and mounted in Roti-Histokitt (Roth, Germany). Microphotographs were obtained with an Eclipse E800 microscope (Nikon, France). Counts were performed by an investigator who was blinded to animal genotypes using National Institutes of Health IMAGE version 1.62 software (USA).

The SDH staining was performed as follows: freshly dried cryostat sections (14 μm) were incubated for 5 min at room temperature (19–21°C) in a medium composed of 2 ml of nitroblue tetrazolium stock solution (100 ml phosphate-buffered saline at pH 7.6, 6.5 mg KCN, 185 mg EDTA, 100 mg NB), 0.2 ml succinate stock solution (2.7 g sodium succinate, 20 ml distilled water), and 0.7 mg phenazine methosulfate. The sections were rinsed in acetone gradient, cleared, and mounted in glycerin jelly. Microphotographs were obtained with an Eclipse E800 microscope (Nikon). Muscle fiber cross-sectional area of SDH-positive oxidative fibers and SDH-negative glycolytic fibers was determined from digitized muscle sections, using ImageJ software (http://imagej.nih.gov). The cross-sectional area of 50 individual fibers/category and mouse was determined from the number of pixels within manually outlined fiber boundaries by an investigator who was blinded to genotypes and treatment groups.

### Statistical analysis

No previous statistical calculation was employed to determine sample size. Instead, sample size in our experiments was chosen based on usual procedures and best practices in the field (Ludolph *et al*, 2010). Unless otherwise indicated, data are expressed as the mean ± SEM. For each figure, statistical tests are justified as appropriate. The significance of the difference between two groups was determined with a two-tailed Student's *t*-test. Multiple comparisons were analyzed by analysis of variance (ANOVA) followed by Fisher's least significant difference (Fisher's LSD) *post hoc* test. For axotomy experiments, paired *t*-tests were performed to compare between muscle ipsilateral and contralateral to the crush for each mouse. Statistical tests used and the number of samples or animals are specified in the respective figure legends. Differences with *P*-values ≤ 0.05 were considered significant. Statistical analysis was performed using Graphpad Prism version 6.0e (GraphPad, USA).

**The paper explained**

**Problem**

Amyotrophic lateral sclerosis is the most common adult motor neuron disorder characterized by motor neuron death and muscular atrophy, which ultimately leads to death. To date, there is no efficient therapy for this fatal disease. ALS has a very important metabolic component as, in patients, energy deficit negatively impacts disease progression and correlates with poorer prognosis. Muscle denervation in disease progression seems to be a dynamic process of denervation and reinnervation that is different for the different muscle fiber types, with the glycolytic muscle fibers being the first affected. In the present work, we investigated potential metabolic mechanisms that may be coupled to the muscle–nerve dialogue and NMJ stability and that may account for the metabolic imbalance described in patients.

**Results**

The most important finding of our study in this neuromuscular disease is an increase in endurance capacity during prolonged oxidative exercise associated with a switch in fuel preference in glycolytic muscle fibers toward lipid oxidation, prior to motor symptoms. The increased expression of genes involved in lipid handling is accompanied by a decrease in expression and activity of PFK1, the rate-limiting enzyme of glycolysis. Glycolysis inhibition is a bottom-to-top process, orchestrated by PDK4, a mitochondrial enzyme that blocks the entry of pyruvate, the end product of glycolysis, into the TCA cycle. The increase in lipid use is not accompanied by an increase in mitochondrial biogenesis, but is associated with mitochondrial deficit and increased oxidative stress. We further show that it is possible to pharmacologically modulate this pathway and restore metabolic equilibrium. By doing so, we were able to prevent denervation and muscular atrophy and improve metabolic profile in our ALS mouse model.

**Impact**

We show for the first time that muscle tissue suffers metabolic alterations early in the course of disease development, alterations that influence the course of the pathology. These alterations are specific to glycolytic muscle fibers and therefore can explain the sensitivity of glycolytic fibers to atrophy and denervation. By exploring this mechanism, we pinpoint the molecular components involved in these modifications. We identify a potential therapeutic target in this pathway, which, if targeted, may improve prognosis and quality of life for ALS patients.

## Supporting Information

Supplementary Figures S1–S7 and Supplementary Table S1

Review Process File
