# Supplementary material for: A metabolic switch toward lipid use in glycolytic muscle is an early pathologic event in a mouse model of amyotrophic lateral sclerosis
Source: EMBO Mol Med. 2015 Mar 27;7(5):526–46. doi: 10.15252/emmm.201404433 (PMC4492815; doi:10.15252/emmm.201404433)
Supplement: Supplementary file 1 [file emmm0007-0526-sd1.pdf]

SUPPLEMENTAL INFORMATIONS

TABLE OF CONTENT

Supplemental table S1: Sequences of primers used for qPCR.

Figure S1: Early changes in glucose handling in SOD1^{G86R} mice.

Figure S2: Gene expression changes in *tibialis anterior* of SOD1^{G93A} mice during disease progression.

Figure S3: *Pdk4*, *Pdk2* and *Foxo1* expression in *soleus* of WT and SOD1^{G86R} mice.

Figure S4: *Pdk4* expression is increased in *deltoïd muscle of ALS patients*.

Figure S5: NMJ morphology and gene regulation in TA after sciatic nerve crush.

Figure S6: Muscle gene regulation after sciatic nerve axotomy.

Figure S7: Effects of DCA treatment on body weight and muscle characteristics.

Gene	ID	5'-3'	3'-5'
Acetylcholine receptor α	*AChR α*	CCA-CAG-ACT-CAG-GGG-AGA-AG	AAC-GGT-GTGTGT-TGA-TG
Acetylcholine receptor γ	*AChR γ*	GAG-AGC-CAC-CTC-GAA-GAC-AC	GAC-CAA-CCT-CAT-CTC-CCT-GA
Acyl-CoA synthetase family member 2	*Acsf2*	CTC-TTT-CCC-ACC-ACA-ACA-TCG	TCT-GCA-GTC-TTT-GTG-GGC-A
Atrogin1/F-box only protein 32	*Atg-1/ Fbxo32*	AGT-GAG-GAC-CGG-CTA-CTG-TG	GAT-CAA-ACG-CTT-GCG-AAT-CT
Carnitine palmitoyl transferase 1	*Cpt-1β*	GGC-TCC-AGG-GTT-CAG-AAA-GT	TGC-CTT-TAC-ATC-GTC-TCC-AA
Citrate synthase	*Cs*	TAG-CAA-ATC-AGG-AGG-TGC-TTG-T	TCT-GAC-ACG-TCT-TTG-CCA-AC
CyclophilinA	*Cypa*	CTG-GTT-GCT-GAT-GGT-GGT-TA	CTT-CCC-AAA-GAC-CAC-ATG-CT
Cytochrome c oxidase subunit 1	*Cox1*	TCC-ACT-ATT-TGT-CTG-ATC-CGT-ACT	AGT-AGT-ATA-GTA-ATG-CCT-GCG-GCT-A
Estrogen related receptor α	*Errα*	CCT-GGT-CGT-TGG-GGA-TGT	GGA-CAG-CTG-TAC-TCG-ATG-CTC
Fatty acid translocase/CD36 antigen	*CD36*	ATT-AAT-GGC-ACA-GAC-GCA-GC	TTC-AGA-TCC-GAA-CAC-AGC-GT
Forkhead box O1	*Foxo1*	GTG-AAC-ACC-AAT-GCC-TCA-CAC	CAC-AGT-CCA-AGC-GCT-CAA-TA
Glutathione peroxidase 1	Gpx1	CAC-CCG-CTC-TTT-ACC-TTC-CT	TCG-ATG-TCG-ATG-GTA-CGA-AA
Lipoprotein lipase	*Lpl*	GGG-CTC-TGC-CTG-AGT-TGT-AG	CCA-TCC-TCA-GTC-CCA-GAA-AA
Mitofusin 2	*Mfn2*	CGA-GGC-TCT-GGA-TTC-ACT-TC	CAA-CCA-GCC-AGC-TTT-ATT-CC
Muscle specific ring finger protein1	*MuRF1/ Trim63*	GCA-GGA-GTG-CTC-CAG-TCG	TCT-TCG-TGT-TCC-TTG-CAC-AT
Nuclear respiratory factor 1	*Nrf1*	TGG-AGT-CCA-AGA-TGC-TAA-TGG	GCG-AGG-CTG-GTT-ACC-ACA
Peroxisome proliferator-activated receptor β/δ	Ppar β/δ	ATG-GGG-GAC-CAG-AAC-ACA-C	GGA-GGA-ATT-CTG-GGA-GAG-GT
Peroxisome proliferator-activated receptor γ coactivator 1α	*PGC1α*	TGC-TGC-TGT-TCC-TGT-TTT-C	CCC-TGC-CAT-TGT-TAA-GAC-C
Phosphofructo-kinase 1	*Pfk 1*	GCC-AAA-GGT-CAG-ATT-GAG-GA	CAG-GTT-CTT-CTT-GGG-GAG-AGT
Pyruvate dehydrogenase kinase 2 (mouse)	*Pdk 2*	TTC-AGC-AAT-TTC-TCC-CCG-TC	AGG-CAT-TGC-TGG-ATC-CGA-AG
Pyruvate dehydrogenase kinase 2 (human)	*Pdk 2*	GTT-CCT-GGA-CAA-GGA-TCC-CG	TGT-ACT-CAA-GCA-CGC-CTT-GT
Pyruvate dehydrogenase kinase 4 (mouse)	*Pdk 4*	GCT-GGA-TGT-TTG-GTG-GTT-CT	TGC-TTT-GAT-TCC-TCC-CAT-CC
Pyruvate dehydrogenase kinase 4 (mouse)	*Pdk 4*	CCT-GTG-AGA-CTC-GCC-AAC-AT	TCC-ACC-AAA-TCC-ATC-AGG-CT
Ribosomal protein, large, P0 (human)	RPLP0	TGG-CAG-CAT-CTA-CAA-CCC-TG	ATC-TGC-AGA-CAG-ACA-CTG-GC
RNA Polymerase II polypeptide A	*Polr2a*	AAT-CCG-CAT-CAT-GAA-CAG-TG	CA-TCC-ATT-TTA-TCC-ACC-ACC
TATA box binding protein	*Tbp*	CCA-ATG-ACT-CCT-ATG-ACC-CCT-A	CAG-CCA-AGA-TTC-ACG-GTA-GAT

Table S1: Sequences of primers used for qPCR.

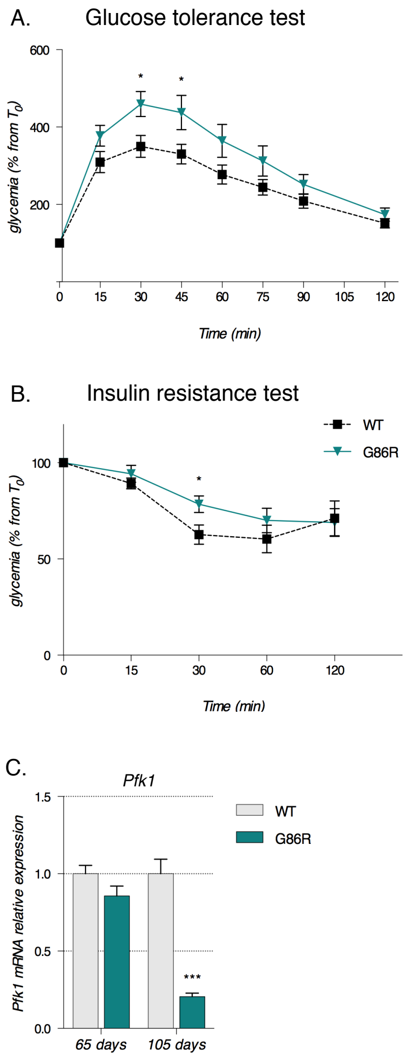

Figure S1. Early changes in glucose handling in SOD1^{G86R} mice.

A: A glucose tolerance test was performed on 65 day old mice after 18 hours of fasting. Blood levels of glucose were measured at several time points following glucose administration. Each point represents mean ± SEM of percentage from initial blood glucose concentration at T_0 over time. The significant differences observed correspond to P=0.032 at 30 minutes and P=0.038 at 45 minutes after glucose administration (n=7/genotype, multiple *t* tests).

B: An insulin tolerance test was performed on 65 day old mice after 4 hours of fasting. Blood levels of glucose were measured at several time points following insulin administration. Values represent percent from initial blood glucose concentration at T_0 over time ± SEM. The response to insulin presented a significant difference between the two groups at T_{30} post-injection with P =0.034, (n=6 and 9 for WT and SOD1^{G86R} respectively, multiple *t* tests).

C: Relative mRNA levels of phosphofructokinase 1 (*Pfk1*) were measured by qPCR at the indicated ages (65 and 105 days) in *tibialis anterior* of WT and SOD1^{G86R} mice. Graphs represent mean fold change ± SEM from age-matched WT. ***P<0.0001 (n=6 and 8 for WT and SOD1^{G86R} respectively at 65 days, n=5 and 6 for WT and SOD1^{G86R} respectively at 105 days, two way ANOVA followed by Fisher's LSD *post hoc* test).

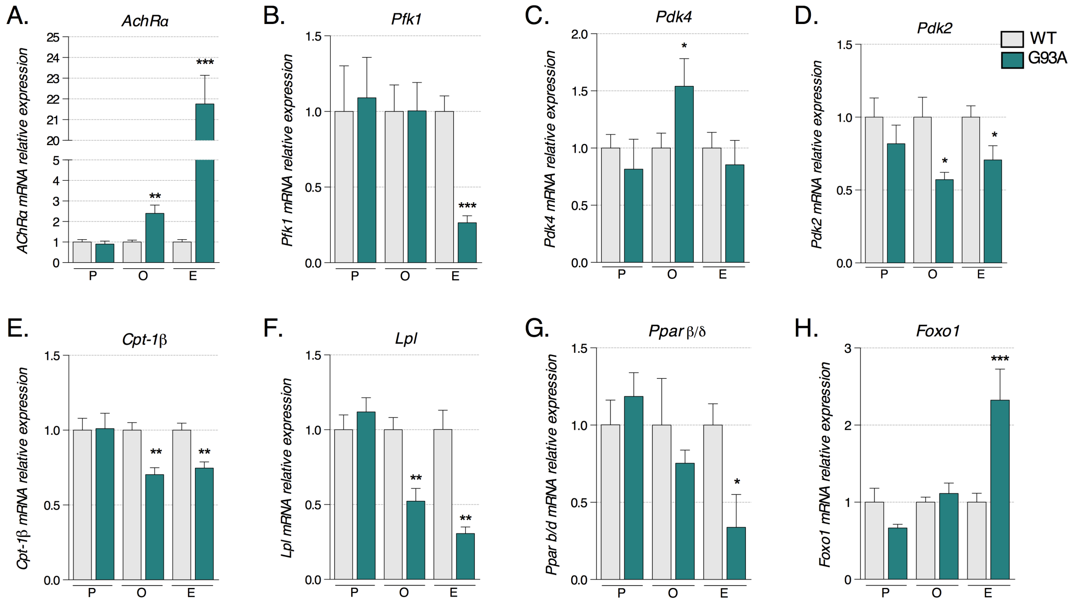

Figure S2: Gene expression changes in *tibialis anterior* of SOD1^{G93A} mice during disease progression.

Relative mRNA levels of *AChRα* (A), *PfK1* (B) *Pdk4* (C), *Pdk2* (D) *Cpt1-β* (E), *Lpl* (F), *Pparβ/∂*, (G) *Foxo1* (H) were measured by qPCR in TA from SOD1^{G93A} mice (G93A) and wildtype (WT) littermates at three stages of the disease: (P) from 30 to 36 days of age a presymptomatic stage, (O) from 63 to 75 days of age corresponding to the onset of the disease when mice develop signs of hindlimb weakness, and (E) from 150 to 175 days of age the end stage of the disease when mice display hindlimb weakness, hindlimb paralysis or loss of the righting reflex (Ngo *et al,* 2012). Graphs represent mean fold change ± SEM from age-matched WT. **P=0.015 and ***P<0.0001 for *AChRα*, ***P<0.0001 for *Pfk1*, *P=0.0284 for *Pdk4*, *P=0.015 at O and 0.0401 at E for *Pdk2*, **P=0.0019 at O and **P=0.0033 at E for *Cpt-1β,* **P=0.0032 at O and P=0.0031 at E for *Lpl,* *P=0.0313 for *Pparβ/δ and* ***P<0.0001 for *Foxo1* (n=5, Student's *t* test).

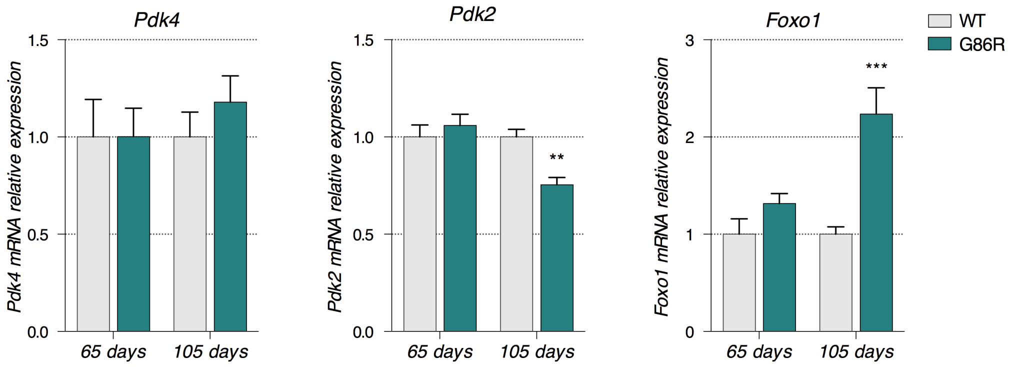

Figure S3: *Pdk4*, *Pdk2* and *Foxo1* expression in *soleus* of WT and SOD1^{G86R} mice.

Relative mRNA levels of *Pdk4* (left panel), *Pdk2* (middle panel) and *Foxo1* (right panel) were measured by qPCR in *soleus* at the indicated ages. Graphs represent mean fold change ± SEM from age-matched WT. *P*-values *vs* WT: P*dk2* *P*=0.0023 at 105 days; *Foxo1* *P*<0.0001 at 105 days (n=7 and 6 for WT and SOD1^{G86R} respectively at 65 days, n=6 and 7 for WT and SOD1^{G86R} respectively at 105 days, two way ANOVA followed by Fisher's LSD *post hoc* test).

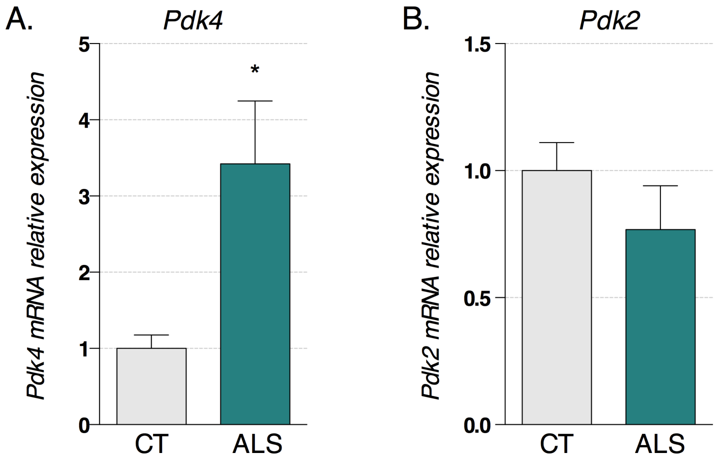

Figure S4: *Pdk4* expression is increased in *deltoid muscle of ALS patients*.

Relative mRNA levels of *Pdk4* (left panel) and *Pdk2* (right panel) were measured by qPCR in *deltoid* from controls (CT) or patients with definite ALS (ALS). Graphs represent mean fold change ± SEM from CT. *P*-values *vs* CT: 0.0352 (CT: n=7, ALS: n=11, Student's *t* test).

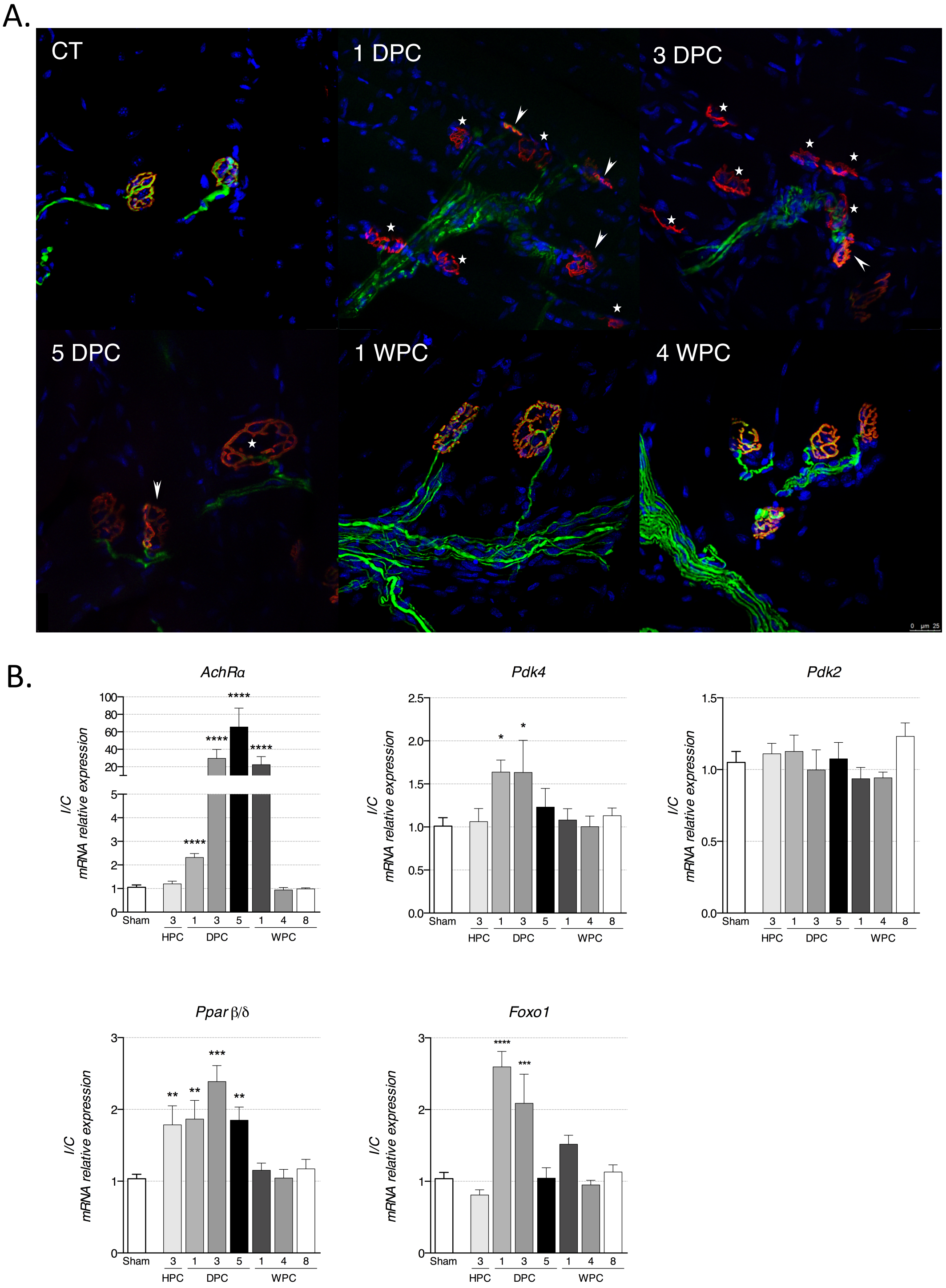

Figure S5: NMJ morphology and gene regulation in *tibialis anterior* (TA) after sciatic nerve crush.

A: Confocal micrographies of neuromuscular junctions (NMJ) in TA from control mice (CT) or after sciatic nerve crush. Mice were sacrificed 1, 3 and 5 days post-crush (DPC) and 1 and 4 weeks post-crush (WPC). Axons are constitutively labeled with YFP (green) in Thy1-YFP mice, while TRITC-bungarotoxin (αBGT) labeled acetylcholine receptors (red) delineate motor endplates. In CT, motor endplates are singly innervated by terminal axons. At 1DPC, some motor endplates already show a decrease of YFP labeling (stars) while others (arrowheads) are comparable to CT. The number of motor endplates fully denervated increases at 3DPC. From 5 DPC, reinnervation becomes visible and contacts between axons and endplates are re-established at 1 week although YFP labeling remains lower than in CT. NMJ present with normal morphology at 4 WPC.

B: Relative mRNA levels of the denervation markers *AChRα*, *Pdk4*, *Pdk2, Pparβ/δ* and *Foxo1* were measured by qPCR in TA after sciatic nerve crush. For each gene, the expression level in the muscle ipsilateral (I) to the crush is normalized with the contralateral (C) muscle which serves as internal control. The graphs represent the mean of I/C ±SEM. P-values for crush *vs* sham. *AchRα* ****P<0.0001*; Pdk4* *P=0.0159 and 0.0200 at 1 and 3DPC; *Pparβ/δ*: **P=0.0042, 0.0017 and 0.0040 at 3 hours post-crush (3HPC), 1 and 5DPC and *** P<0.0001 and *Foxo1*: ****P<0.0001 and *** P=0.0002; (n=8/time point except for 5DPC with n=7, one way ANOVA followed by Fisher's LSD *post hoc* test).

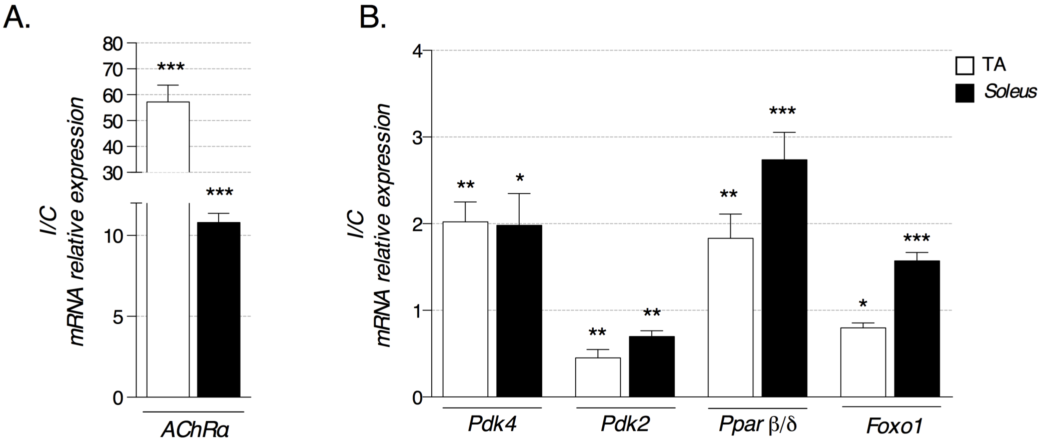

Figure S6: Muscle gene regulation after sciatic nerve axotomy.

Relative mRNA levels of the denervation marker *AChRa* (A) and *Pdk4*, *Pdk2, Pparβ/δ* and *Foxo1* (B) were measured by qPCR in TA and *soleus* two weeks after axotomy. For each gene, the expression level in the muscle ipsilateral (I) to the axotomy is normalized with the contralateral (C) muscle which serves as internal control. The graphs represent the mean of I/C ±SEM. P-values for I *vs* C. *AchRα* ***P<0.0001 in TA and *soleus; Pdk4* ** P=0.0011 and * P=0.0266; *Pdk2* ** P=0.0013 (TA) and 0.0028 (*Soleus*); *Pparβ/δ*: **P=0.0219 and *** P<0.0001; *Foxo1*: *P=0.0209 and *** P=0.0008 (n=8, Paired Student's *t* test).

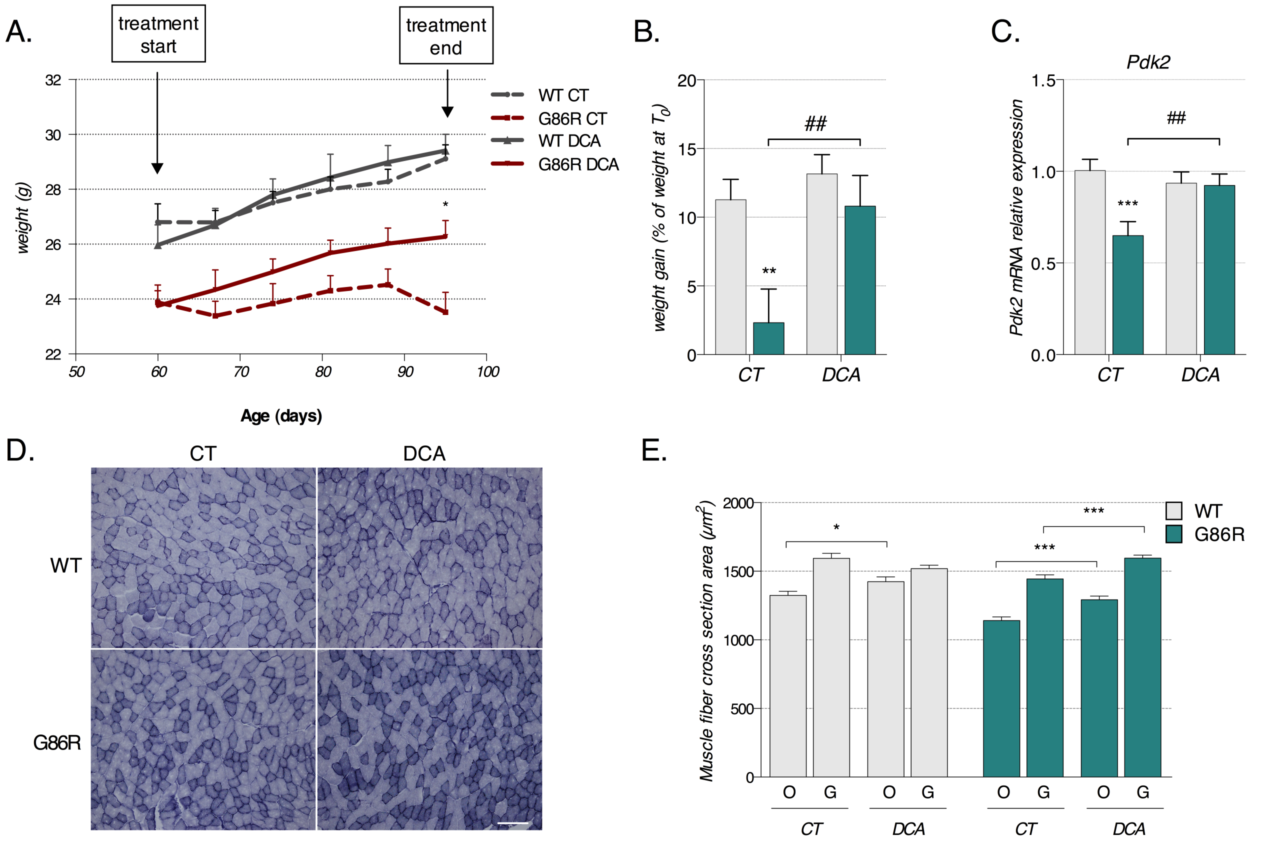

Figure S7: Effects of DCA treatment on body weight and muscle characteristics.

A: Left: Weight over time in WT and SOD1^{G86R} animals in CT and DCA treated groups. Each point represents mean weight ± SEM at a given time point for a given group. At week 5 of treatment *P=0.011 (n=9/genotype in CT groups, n=9 and 8 for WT and SOD1^{G86R} respectively in DCA group, multiple t test between SOD1^{G86R} CT vs DCA).

B: Weight gain at the end-point of treatment expressed means ± SEM of percent from T_0; **P=0.0013 between WT CT and SOD1^{G86R} CT groups, and $^{\#\#}P$=0.0050 between SOD1^{G86R} CT and SOD1^{G86R} DCA (n=9/genotype in CT groups, n=9 and 8 for WT and SOD1^{G86R} respectively in DCA group, two way ANOVA followed by Fisher's LSD *post hoc* test).

C: Relative transcript levels of P*dk2* in WT and SOD1^{G86R} animals in CT and DCA treated groups. Graphs represent mean fold change ± SEM from age-matched CT WT. ***P=0.0005 between WT CT and SOD1^{G86R} CT groups, and $^{\#\#}P$=0.0069 between SOD1^{G86R} CT and SOD1^{G86R} DCA (n=9/genotype in CT groups, n=9 and 8 for WT and SOD1^{G86R} respectively in DCA group, two way ANOVA followed by Fisher's LSD *post hoc* test).

D: Representative microphotographs of SDH activity in TA cross-sections of WT and SOD1^{G86R} mice at 95 days in CT and DCA treated groups. Oxidative fibers appear purple while glycolytic fibers appear white. Scale bar: 100μm.

E: Measure of cross-section area of oxidative (O) and glycolytic (G) fibers in TA of WT and SOD1^{G86R} mice at 95 days in CT and DCA treated groups. Graphs represent mean area ± SEM. *P=0.0174 between O-WT CT and O-WT DCA groups, and $^{***}P$=0.0003 and 0.0004 between respectively O-SOD1^{G86R} CT and O-SOD1^{G86R} DCA and G-SOD1^{G86R} CT and G-SOD1^{G86R} DCA (n=5, two way ANOVA followed by Fisher's LSD *post hoc* test).
