# Supplementary material for: A metabolic switch toward lipid use in glycolytic muscle is an early pathologic event in a mouse model of amyotrophic lateral sclerosis
Source: EMBO Mol Med. 2015 Mar 27;7(5):526–46. doi: 10.15252/emmm.201404433 (PMC4492815; doi:10.15252/emmm.201404433)
Supplement: Supplementary file 2 [file emmm0007-0526-sd2.pdf]

A metabolic switch towards lipid use in glycolytic muscle is an early pathologic event in a mouse model of Amyotrophic Lateral Sclerosis

Lavinia Palamiuc, Anna *Schlagowski*, Shyuan T. Ngo, Aurelia Vernay, Sylvie Dirrig-Grosch, Alexandre Henriques, Anne-Laurence Boutillier, Joffrey Zoll, Andoni Echaniz-Laguna, Jean-Philippe Loeffler, Frédérique René

Corresponding authors: Frédérique René and Jean-Philippe Loeffler, Université de Strasbourg-INSERM

Review timeline:

Submission date:	14 July 2014
Editorial Decision:	28 August 2014
Revision received:	23 December 2014
Editorial Decision:	09 February 2015
Revision received:	17 February 2015
Accepted:	20 February 2015

Transaction Report:

(Note: With the exception of the correction of typographical or spelling errors that could be a source of ambiguity, letters and reports are not edited. The original formatting of letters and referee reports may not be reflected in this compilation.)

Editor: Céline Carret

1st Editorial Decision 28 August 2014

Thank you for the submission of your manuscript to EMBO Molecular Medicine. We have now heard back from the three referees whom we asked to evaluate your manuscript. Although the referees find the study to be of potential interest, they also raise a number of concerns that need to be specifically addressed in the next version of the manuscript.

As you can see from the reports below, while referee 3 is supportive of publication, referees 1 and 2 are much more reserved and list several serious issues that preclude publication of the article in this form. Referees 1 and 2 have a common important concern regarding the limited provided evidence to rule out a role for denervation in the observed metabolic switch. Referee 1 in addition, comments about the use of this particular SOD1 mutant model for ALS, the lack of drug effect analysis in terms of survival and time-dependency and the limited mechanistic insights. Given the interest of the topic however, we would be happy to consider a revision of your manuscript if you can address the issues that have been raised in the reports and especially the points I have highlighted. Please note that it is EMBO Molecular Medicine policy to allow only a single round of revision and that, as acceptance or rejection of the manuscript will depend on another round of review, your responses should be as complete as possible.

EMBO Molecular Medicine has a "scooping protection" policy, whereby similar findings that are published by others during review or revision are not a criterion for rejection. Should you decide to

submit a revised version, I do ask that you get in touch after three months if you have not completed it, to update us on the status.

Please also contact us as soon as possible if similar work is published elsewhere. If other work is published we may not be able to extend the revision period beyond three months.

I look forward to seeing a revised form of your manuscript as soon as possible.

***** Reviewer's comments *****

Referee #1 (Comments on Novelty/Model System):

Only one mouse model of SOD1 mutation, which is rarely used in the field.

Referee #1 (Remarks):

The manuscript by Palamiuc and colleagues investigates the metabolic changes in the skeletal muscle of the G86R SOD1 mutant mouse model of familial ALS. They detect an array of changes compatible with a shift of metabolism from glucose utilization to preferential lipid utilization in glycolytic muscle fibers (i.e., the TA muscle). These changes are detected at 65 days of age, prior to the onset of overt denervation and paralysis. They also detect an increase in several enzymes involved in lipid utilization in mitochondrial beta-oxidation. PDK4 is increased in parallel with the decreased glucose utilization, increased glycogen accumulation, and increased lipid utilization. The administration of DCA (500 mg/Kg/day) starting at day 65 in the drinking water resulted in reversal of several parameters of the switch at day 95, accompanied by preservation of body weight and muscle strength, presumably by inhibiting PDK activity. Based on these observations, they propose that the hypermetabolism and paradoxical weight loss observed in the majority of ALS patients could be related to this metabolic switch in glycolytic muscle fibers and that this could be a therapeutic target.
This work builds on several previous reports by the same and other groups of the possible involvement of skeletal muscle in ALS. This has been a controversial issue, especially in the case of the SOD1 mice. Some reports have discounted this effect by showing essentially no effects of knocking out mutant SOD1 selectively from muscle. Others have shown the opposite, by generating muscle specific expression of mutant SOD1 in muscle. PGC1alpha overexpression in muscle increased strength and muscle trophism in mutant SOD1 mice, without extending their life. This work seeks to shed new light on the potential involvement of muscle, focusing on metabolic changes.
The fact that type II fibers are more prone to degenerate in ALS (sporadic and familial, both in mice and men) has been known for a long time, but the causes have been largely attributed to preferential and more rapid degeneration of type 2 spinal cord motor neurons. It appears that this work suggests that the problem may be intrinsic to the muscle cells, where a switch towards lipid utilization due to inhibition of glucose oxidation could be associated or even predispose to a demise of the NMJ. This line of thinking would support the use of DCA to stimulate pyruvate utilization and restore glucose oxidation.
Overall, this manuscript expands our understanding of muscle involvement in the SOD1 mice. It highlights some interesting aspects of metabolism alterations and strengthens the concept that ALS is a systemic disorder, not exclusively limited to motor neurons. There are some limitations in the study.
1) The main limitation is probably the lack of mechanistic interpretation of the causes behind the metabolic switch. Is mutant SOD1 directly responsible, and if so how?
2) Also problematic is the demonstration that the process is intrinsic to skeletal muscle. The lack of overt denervation does not exclude that the synapses may be dysfunctional and that the problem originates in the type 2 motor neurons, which innervate predominantly fast twitch muscles. The denervation by axotomy does not necessarily prove the point because of the different fiber types. In addition, the effects of the axotomy on the soleus are not described.
Other points:
3) The mouse strain utilized is the one originally developed by Gordon in 1995. This mouse

expresses G86R mutant murine SOD1. It develops a very fast disease phenotype with death earlier than any of the other more commonly used lines, such as the G93A and G37R. It would be important to know if the metabolic changes described in the skeletal muscle in this mouse also apply to the G93A (wild-type like) mutant mouse.
4) There are no survival outcomes described here, in untreated mutant mice and in association with DCA treatment. Only one time point for muscle strength is shown, presumably at 95 days, end of treatment. Not enough information about disease course (onset, progression, and survival) is provided to accurately evaluate the efficacy of the treatment.
5) There are really no hypotheses proposed for the interpretation of the relationship between the switch and mitochondrial dysfunction. Which comes first and why?
6) The demonstration of the mitochondrial recovery upon DCA is not "functional" as defined in the discussion of the manuscript. Protein and mRNA levels do not necessarily define a functional state.
7) The number and volume of the different types of muscle fibers pre and post treatment are not directly measured. Thus, it is unclear if some of the metabolic changes observed can be attributed to fiber type switch at different stages during the course of the disease.
8) The pathogenic role of the switch is proposed to be associated with increased ROS from beta-oxidation derived NADH, which could damage mitochondria. However, there is no direct evidence supporting this interpretation.
9) An explanation of the meaning of the different markers of denervation used would be helpful.

Referee #2 (Comments on Novelty/Model System):

The authors have provided an extremely convincing demonstration of the metabolic switch from glucose to lipids in a glycolytic muscle in a model of ALS. Several of the senior authors in this manuscript have published prior data suggesting the important role of muscle metabolic changes in ALS models as independent of motor axon alterations, or even as a possible initiator of motor axon and motor neuron changes. The present manuscript can stand on its own following elimination of all statements that the metabolic switch is due to denervation. If they really believe that denervation (even partial denervation) is not playing a role, they need to present cogent data to support those statements. The present manuscript does not contain such, and therefore needs to be substantially revised if it is to be considered for acceptance..

Referee #2 (Remarks):

The manuscript by Palamiue provides compelling data on the metabolic switch towards lipid use in the tibialis anterior, a glycolytic muscle, in presymptomatic G86R mSOD mice, a model of ALS. The mechanism is potentially explained by the demonstration of increased expression of PDK4 and inhibition of PFK1, resulting in the switch of fuel preference from glucose to lipids. Dichloroacetate delayed symptom onset and improved mitochondrial dysfunction.
The data are very convincing. However, a major concern is the implication that all the documented changes appear to be an independent effect in muscle and are not the result of denervation or changes in axon terminals or the motor neuron. Their sole evidence that the metabolic switch is not dependent on denervation, either complete or partial, is in Fig E2. Following axotomy of WT mice, PDK changes are purportedly different, Furthermore, at 65 days the changes in ACHR subunits expected following denervation (Fig 2) are not seen. Yet we are not shown any other data following denervation of WT mice.. Perhaps nerve crush rather than axotomy might be a more meaningful experiment (see paper by Magill et al (Exp Neurol 207:64-74, 2007). In order to conclude that the metabolic switch is not due to altered information from the motor neuron, they have the obligation to compare much of their RNA expression data between G86R mice and WT subjected to sciatic nerve crush injury. Clearly their data on the metabolic switch is sufficiently compelling to stand on its own. But in the absence of appropriate WT sciatic nerve crush data, they can not conclude that denervation, either partial or complete, plays no role. As they acknowledge, DCA therapy can improve motor neuron function by effects on astrocytes, thus DCA does not necessarily provide evidence for muscle-independent beneficial effects.

Referee #3 (Comments on Novelty/Model System):

The manuscript titled "A metabolic switch towards lipid use in glycolytic muscle is an early pathologic event in a mouse model of Amyotrophic Lateral Sclerosis.," by Lavinia Palamiuc, Anna Schlagowski, Aurelia Vernay, Sylvie Grosch, Anne Laurence Boutillier, Joffrey Zoll, Shyuan T. Ngo, Jean-Philippe Loeffler and FrÈdÈrique RenÈ address a yet unknown metabolic alteration in the muscle tissue of the murine model of ALS. The authors describe a change in glycolytic muscle metabolism that specifically alters muscle metabolic plasticity in glycolytic fibers. They found that these metabolic alterations occur very early in the course of the disease, before the onset of neuromuscular junctions denervation, and prior to motor neuron death.
This work greatly contributes to clarify the metabolic alterations described in ALS and demonstrates that by improving metabolic function through DCA, it is possible to improve motor function, maintain muscular integrity and delay denervation.
This information is relevant to understand and unravel the pathology of ALS and contribute to design new therapeutic strategies.
The article is very well written and illustrated with a comprehensive art work.
I highly recommend its publication without modifications.

1st Revision - authors' response 23 December 2014

REFEREE 1:

1) "Is mutant SOD1 directly responsible, and if so how? "
Here our answer is clearly no. We provide evidence for this argument through our analysis of muscle biopsies obtained from patients with SPORADIC ALS. There was no familial history for these patients and hence no obvious genetic cause (including the absence of any SOD1 mutations). Consequently changes in metabolic flexibility (governed by PDKs) are specific to ALS and not SOD1 mutations. These data (specific increase of PDK4 but not PDK2 in the skeletal muscle of ALS patients) are now presented as supplemental material (Figure E4) in the manuscript and discussed (results page 6 paragraph 3 last sentence and discussion page 12 lines 4-7).

2) " the effect of axotomy on the soleus is not described "
This information is now provided. Results are presented page 7 first paragraph and Figure E6 and discussed in the last paragraph on page 12. As shown, the *soleus* behaves essentially as the *tibialis anterior* in this experimental procedure.
Further, as suggested by the referee (and by referee 2) we have performed a detailed "Nerve crush" study with a kinetic from 3 hours to 8 weeks to produce a transient synaptic dysfunction followed by denervation and reinnervation. The results are presented page 7, first paragraph and Figure E5 and discussed on page 13 in the first paragraph with the axotomy. This study shows that PDKs are modified in the initial phase of the experiment (although to a lesser extent than in the ALS model) and then return to normal levels long before functional motor restoration and recovery of a normal neuro-muscular junction (as shown by NMJ histology and AChRa mRNA expression recovery)

3) "It would be important to know if the metabolic changes described in the skeletal muscle in this mouse also apply to the G93A (wild-type like) mutant mouse."
We have obtained tissues from the mouse line overexpressing human *SOD1* with the G93A mutation (B6.Cg-Tg(SOD1-G93A)1Gur/J) and analyzed the expression of several relevant genes in TA at three stages based on motor behavior: a presymptomatic stage (30 to 36 days of age), the onset of disease when mice develop signs of hindlimb weakness ((63 to 75 days of age) and the end-stage when mice display hindlimb weakness, hindlimb paralysis or loss of the righting reflex (150 to 175 days of age) (Ngo *et al,* 2012). As mentioned in the discussion "In $SOD1^{G93A}$ mice, although induction of *Pdk4* expression is detected at the onset, the global metabolic profile of the mice differs from the one found in $SOD1^{G86R}$ mice. However, the difference can rely on the profound mitochondrial alterations described in the $SOD1^{G93A}$ mice as early as post-natal day 30 and age largely documented in the literature".
The data are presented all along the result section and in Figure E2 as supplemental material and discussed pages 12.

4) This point concerns the DCA treatment and is also raised by referee 2.
The goal of this study was to analyse the metabolic aspects of ALS. The consequences of DCA treatment on the course of the disease were not studied here. We clearly show that the blockade of PDK reverses the inhibition of the metabolic flexibility. The "clinical" consequences are discussed in reference to the existing literature. See for example the paper by Barbeito's group that show the beneficial effects of DCA on a mouse ALS model. To supplement the data, we have included a figure (Figure E7C) showing that DCA treatment induces an increase of muscle fibre size in $SOD1^{G86R}$ mice as compared to untreated $SOD1^{G86R}$ mice. This change could participate to the increased of grip strength found at the end of the treatment.

5) " There are really no hypotheses proposed for the interpretation of the relationship between the switch and mitochondrial dysfunction. Which comes first and why? "
We do not postulate any mitochondrial dysfunction at the presymptomatic stage used in this study (i.e. 65 days) in the $SOD1^{G86R}$ mice when the metabolic switch is already visible. As shown in Figure 5 and 6, genes involved in mitochondria dynamics (mfn2, Nrf1) or function (citrate synthase, Gpx1) as well as the amount of mitochondrial DNA are comparable to wild-type. Thus the bioenergetic defect lies upstream of the mitochondria and is rather a defect in fuel preference (b-oxidation vs glycolysis). The exact origin of the metabolic switch however remains to be elucidated and is not the goal of the present manuscript.

6)) The demonstration of the mitochondrial recovery upon DCA is not "functional" as defined in the discussion of the manuscript. Protein and mRNA levels do not necessarily define a functional state.
We agree with referee 1. We have now removed the word "functional" from the discussion.

7) "The number and volume of the different types of muscle fibers pre and post treatment are not directly measured. Thus, it is unclear if some of the metabolic changes observed can be attributed to fiber type switch at different stages during the course of the disease."
We have performed a histochemical analysis of the diameter of the SDH+ and SDH- fibers after treatment and showed an increase of both fiber types with DCA in SOD1 mice. This increase in size could account for the maintenance of grip strength in these mice. This information is now included in results section page 9 last paragraph and figure E7C, and discussed page 13 first paragraph line 13-14.

8) The pathogenic role of the switch is proposed to be associated with increased ROS from beta-oxidation derived NADH, which could damage mitochondria. However, there is no direct evidence supporting this interpretation.
In an earlier study, we have demonstrated an increase of ROS in muscle of asymptomatic $SOD1^{G86R}$ mice (Halter et al 2012). In the discussion of this manuscript page 13 first paragraph, we present a plausible hypothesis explaining how increased lipid use could account for increased ROS production that will then have a toxic effect on mitochondria (visible at end-stage of the disease). The same hypotheses are presented by Aon et al., (2014). This review is now given in the reference list.

9) An explanation of the meaning of the different markers of denervation used would be helpful.
An explanation of the meaning of the different markers is now given in the manuscript page 5 paragraph 2.

REFEREE 2

The main criticism by referee 2 deals with our apparently very strong claim that the observed metabolic changes are independent of the denervation. It appears that in several analyses these changes precede any visible denervation. We acknowledge the fact that this depends on the sensitivity of the "test" one uses to evaluate denervation. This claim is now more balanced and removed from the text as suggested by this referee.
Data on nerve crush experiments have been included (See also point 2 of referee 1).

REFEREE 3

Referee 3 comments do no call for a further reply.

2nd Editorial Decision 09 February 2015

Thank you for the submission of your revised manuscript to EMBO Molecular Medicine. We have now received the enclosed reports from the referees who were asked to re-assess it. As you will see the reviewers are now globally supportive and I am pleased to inform you that we will be able to accept your manuscript pending the following final amendments:

1) Please introduce the changes recommended by referee 2. Importantly, this referee asks for the statements suggesting that denervation plays no role in initiating the metabolic switch to be removed and make a case for this. Please comply.

Please submit your revised manuscript within two weeks. I look forward to seeing a revised form of your manuscript as soon as possible.

***** Reviewer's comments *****

Referee #2 (Remarks):

The demonstration of a switch in fuel prefence to lipids in glycolytic mutant SOD 1 muscles is convincingly demonstrated. However, their neglect of the possible role of denervation in inititating such changes is of concern. The authors note in their response to Referee 1, point 5 that the "exact origin of the metabolic switch however remains to be elucidated and is not the goal of the present manuscript"- yet it could have been initiated by early onset mSOD-mediated axonal dysfunction leading to partial denervation. In their revised manuscript they did carry out a nerve crush study (Supplemental Fig E5) and noted an early increase in Pdk4 following denervation. Thus they have provided evidence that partial denervation could contribute to the metabolic switch. Yet they seem to deny this possibility. Although they claim to have provided a "more balanced" interpretation, they truly have not done so. For example in the Abstract, line 4, the sentence starts "We found that prior to denervation..." and in their conclusion Page 15 lines 5-6, they state that metabolic alterations occur .."before the onset of denervation" and lines 7-8 "glycolytic defect are due to additional events distinct from denervation." A similar statement denying the role of denervation is presented in the Results section of "The Paper Explained"- line 3 -"prior to motor symptoms and denervaion." Clearly the metabolic switch could contribute both to motor symptoms and to denervation, and need not have occured prior to the denervation. As the authors suggest in the response to Referee 2, the role of denervation depends on the sensitivity of the techmiques employed to document such denervation, yet their techniques to document very early changes of denervation are not sufficiently sensitive to rule out motor axon-initiated changes. They chose time periods of Presymptomatic of 30-36 days and Onset at 63 days in the G93A mouse in Supplemental FigE2 yet changes in the motor neuron axonal function have been documented between36-63 days or earlier, and reports have noted changes of denervation during this time period. Thus the claim that the metabolic switch is not dependent on denervation is not supported by their own data. The paper's conclusion would be much more compelling with the conservative statement that early changes of denervation could contribute to early stages of the metabolic switch, which could contribute to further NMJ changes and a self-propagating process. In no sense would this statement detract from the cogency of their elegant metabolic data.
They also should document the results of the crush expeiments on Page 7 as a sepaate paragraph.

Referee #3 (Comments on Novelty/Model System):

As a said in my previous review I believe this manuscript is highly relevant.
I found that authors have satisfactorily answered the reviewer's comments.

2nd Revision - authors' response 17 February 2015

1- CHANGES REQUIRED BY REFEREE 2

Abstract section Page 2 line 4:
"prior to denervation" is replaced by "at 65 days of age"

Results section Page 7:

A new subheading entitled "4. NMJ dysfunction could participate to *Pdk4* up-regulation" is added to present crush and axotomy experiments in a separate paragraph.

Discussion section Page 13:

1st paragraph: the last sentence "However, as it stands, our results do not favor patent denervation as a primary cause of the metabolic switch in ALS." Is removed. We have replaced this sentence by the following sentences: " In the $SOD1^{G93A}$ mice, denervation events have been detected at 30 days of age through a detailed study of the NMJ by electron microscopy (Vinsant *et al,* 2013), and impaired neuromuscular transmission has been detected at 28-42 days of age (Rocha *et al,* 2013), long before the onset of motor symptoms. Here, we showed that, at the same period *Pdk4* mRNA levels are comparable to those found in WT mice. Altogether these data show that NMJ dysfunction or destabilization might participate to the *Pdk4* mRNA induction. "

Discussion section Page 15:

1st paragraph line 3: the end of the sentence "before the onset of denervation and prior to motore neuron death in SOD1G86R mice" is removed. The sentence lines 7-8 telling that "glycolytic defect are due to additional events distinct from denervation." Is now replaced by the two following sentences (lines3-7): " These data, in combination with our sciatic nerve crush and axotomy experiments suggest that glycolytic defects might be due to additional events distinct from denervation. However, early changes of synaptic functionality might contribute to early stages of the metabolic switch, which in turn, could contribute to further NMJ changes and a self-propagating process."

Paper explain Page 22:

Result section line 3: in the sentence "prior to motor symptoms and denervation." denervation is now removed

© EMBO
